# *Vacceed*: a high-throughput *in silico* vaccine candidate discovery pipeline for eukaryotic pathogens based on reverse vaccinology

**DOI:** 10.1093/bioinformatics/btu300

**Published:** 2014-04-29

**Authors:** Stephen J. Goodswen, Paul J. Kennedy, John T. Ellis

**Affiliations:** ^1^School of Medical and Molecular Biosciences, The ithree Institute and ^2^Faculty of Engineering and Information Technology, School of Software, The Centre for Quantum Computation and Intelligent Systems, University of Technology Sydney (UTS), Ultimo, NSW 2007, Australia

## Abstract

**Summary:** We present *Vacceed*, a highly configurable and scalable framework designed to automate the process of high-throughput *in silico* vaccine candidate discovery for eukaryotic pathogens. Given thousands of protein sequences from the target pathogen as input, the main output is a ranked list of protein candidates determined by a set of machine learning algorithms. *Vacceed* has the potential to save time and money by reducing the number of false candidates allocated for laboratory validation. *Vacceed*, if required, can also predict protein sequences from the pathogen’s genome.

**Availability and implementation:** *Vacceed* is tested on Linux and can be freely downloaded from https://github.com/sgoodswe/vacceed/releases (includes a worked example with sample data). *Vacceed* User Guide can be obtained from https://github.com/sgoodswe/vacceed.

**Contact:** John.Ellis@uts.edu.au

**Supplementary information:** Supplementary data are available at *Bioinformatics* online.

## 1 INTRODUCTION

Several subunit vaccines against prokaryotic pathogens have been identified (Ariel *et al.*, 2002; Montigiani *et al.*, 2002; Ross *et al.*, 2001) using reverse vaccinology (Rappuoli, 2000). Vaxign (He *et al.*, 2010) and NERVE (Vivona *et al.*, 2006) are examples of vaccine discovery tools for prokaryotes, but there is currently no equivalent tool for eukaryotes. Freely available bioinformatics tools and an unprecedented volume of –omics data now present an opportunity for *in silico* vaccine discovery for eukaryotic pathogens. A general approach is to use several tools to predict and gather evidence for protein characteristics. From this potential evidence, the researcher makes an informed decision as to a protein’s vaccine candidacy suitability. Determining which tools are appropriate, as well as how to use them, presents the first of many challenges. A further challenge, especially to a researcher with limited programming ability, is to extract and gather the pertinent evidence distributed within large-scale outputs. The subsequent and more imposing challenge is that the evidence is mainly in different formats, contradicting and inaccurate. Poor evidence reliability arises because some of the input data to the tools (e.g. protein sequences and training data) are inaccurate or missing. Moreover, tools used to predict protein characteristics are, in general, inaccurate.

Vaccine candidates identified *in silico* can only be validated in a laboratory. Validation should provide feedback to inform and improve vaccine candidacy decision making. The repetitive nature for this ideal *in silico* approach is in need of automation. Furthermore, an automated process must accommodate an ever-increasing choice of new or improved prediction programs that inevitably replace existing ones.

We have developed *Vacceed* to address the challenges raised here i.e. to provide a flexible, automated process to predict worthy vaccine candidates from large volumes of superfluous, disseminated and noisy data. *Vacceed* is the collective name for a framework of linked bioinformatics programs, Perl scripts, R functions and Linux shell scripts. A previous published study provided guidance in development (Goodswen *et al.*, 2013b).

## 2 DISTINCTIVE FEATURES

A detailed description of all aspects of *Vacceed* is provided in a comprehensive user guide provided as Supplementary Information. The focus of this article is to introduce *Vacceed* via a selection of distinctive features.

**Fig. 1.**
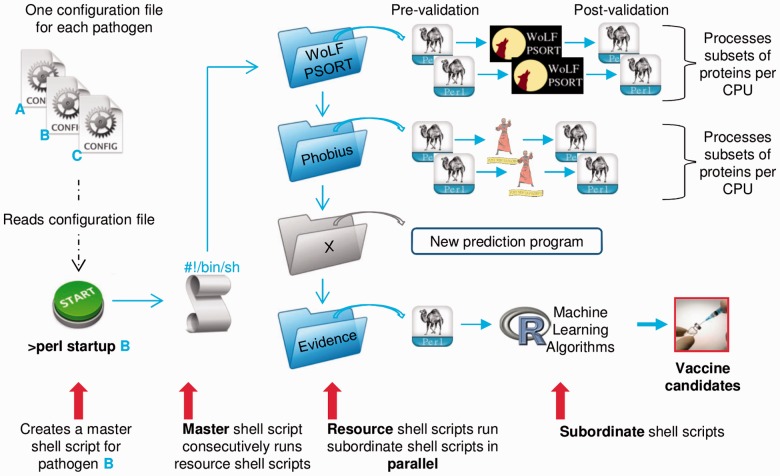
*Vacceed* framework. A set hierarchal structure exists for the execution of all *Vacceed* scripts e.g. startup → master script → resource script → subordinate script (only three resources are shown to maintain clarity)

The *Vacceed* framework is built around the concept of linked resources (see Fig. 1). Each resource, in this context, is built from a central Linux shell script encapsulating all programs needed to perform specific but related tasks. Typical tasks include predicting a particular protein characteristic as well as pre- and post-validation. A resource can be executed as an independent modular unit. This flexible design allows for scalability and easy maintenance. Any prediction program can be integrated within an existing or new resource if it meets the following criteria: runs in a Linux environment, has high-throughput capability, is applicable to eukaryotes, can be trained or has trained data specific to target pathogen and provides consistent text output. From a user’s perspective, all the work involved in the complexity of linking tasks and resources into a seamless continuous pipeline has already been resolved in *Vacceed*. The only time a user must be concerned with the contents of a resource is when adding a new one. There is a template resource script and generic Perl scripts to ease this process.

**Fig. 2.**
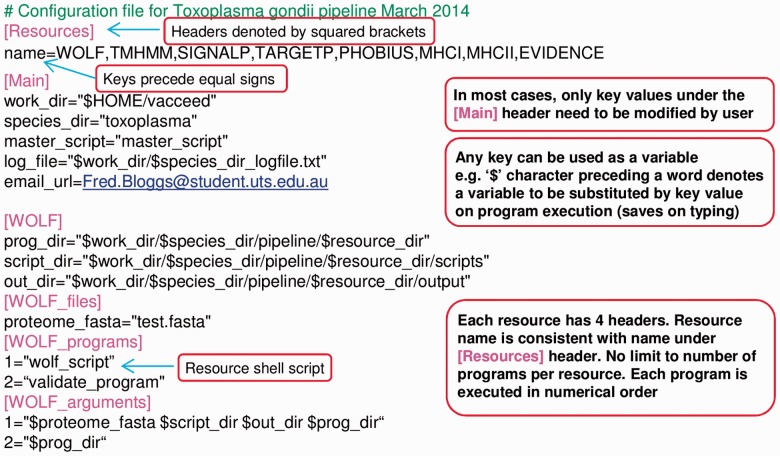
Extract of a *Vacceed* configuration file defined by a header-key format (only one resource, WoLF PSORT, is shown for brevity)

Core to *Vacceed* are user-definable configuration files (see Fig. 2). These files are in effect the user’s interface to configuring each resource, if desired, and consequently controlling the outcome of the entire pipeline. For example, by altering names in a list, the user can determine the resources to be run and their order. The expectation is to have one configuration file for each target pathogen. The command-line syntax to invoke *Vacceed *is ‘perl startup *xx*’, where *xx* determines the appropriate configuration file. Specifying a code allows for multiple instances of *Vacceed* to process different species or resource combinations. No other user input is needed. An e-mail with attached log file is sent on successful completion or immediately following an error.

**Fig. 3.**
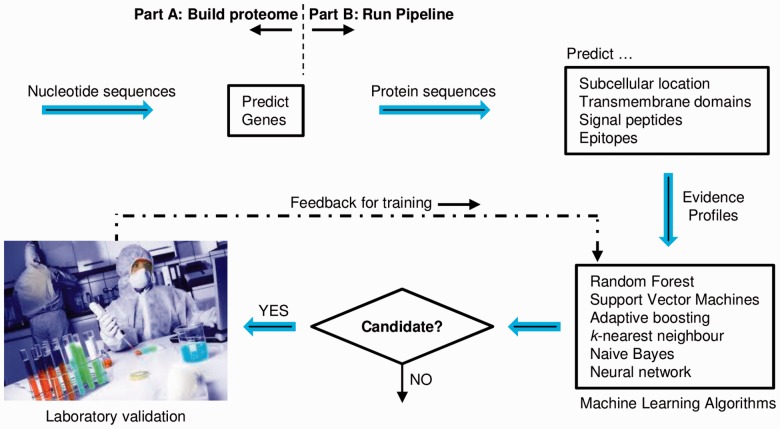
Schematic of data flow in *Vacceed*

The framework is organized into two major parts referred henceforth as part A—build proteome, and part B—run pipeline (see Fig. 3). Table 1 lists the programs currently integrated in each part. A starting prerequisite for part B is a file containing amino acid sequences for proteins from the target eukaryotic pathogen i.e. the proteome. Known protein sequences for many pathogens can be downloaded from public databases. Part A is used, only if required, to predict novel protein sequences and/or collect evidence to support the existence of known proteins. Part A resources typically predict genes, which is one among multiple tasks within linked resources involved in building the proteome. Examples of other tasks are validating gene start and end sequences (e.g. ATG, TAA, TAG or TGA), predicting exon locations relative to gene start, converting predictions to amino acid sequences and homology searching.

**Table 1.** Programs currently integrated in *Vacceed*

Name	Function	URL (last viewed May 2014)
Part A—Build proteome		
Augustus	*Ab initio* gene predictor	http://bioinf.uni-greifswald.de/augustus
GlimmerHMM	*Ab initio* gene predictor	http://ccb.jhu.edu/software/glimmerhmm
BLAT	Aligns expressed sequence tags (ESTs) to DNA	http://genome.ucsc.edu/FAQ/FAQblat.html
GMAP	Aligns expressed sequence tags (ESTs) to DNA	http://research-pub.gene.com/gmap
N-Scan	*Ab initio* gene predictor supported by genome comparison	http://mblab.wustl.edu/software.html
BLASTN	Finds regions of similarity between nucleotide sequences	http://www.ncbi.nlm.nih.gov
BLASTP	Finds regions of similarity between protein sequences	http://www.ncbi.nlm.nih.gov
Part B—Run pipeline (vaccine candidate discovery)		
WoLf PSORT	Protein subcellular localization prediction	http://wolfpsort.seq.cbrc.jp
SignalP	Predicts presence and location of signal peptide cleavage sites	http://www.cbs.dtu.dk/services/SignalP
TargetP	Protein subcellular localization prediction	http://www.cbs.dtu.dk/services/TargetP
Phobius	Combined transmembrane topology and signal peptide predictor	http://phobius.binf.ku.dk/instructions.html
TMHMM	Prediction of transmembrane helices in proteins	http://www.cbs.dtu.dk/services/TMHMM
MHC I-binding	Peptide binding to MHC class I molecules	http://tools.immuneepitope.org/mhci/download
MHC II-binding	Peptide binding to MHC class II molecules	http://tools.immuneepitope.org/mhcii/download

**Fig. 4.**
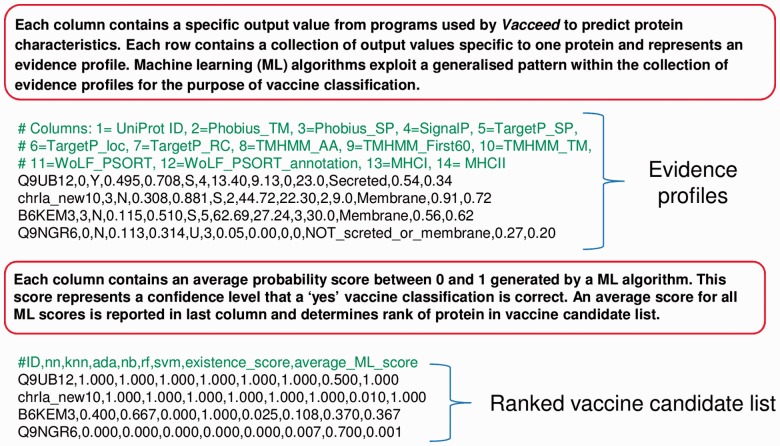
Examples of evidence profiles and a ranked vaccine candidate list (only four proteins out of potentially thousands constituting the target pathogen are shown for brevity)

Part B resources predict protein characteristics. One resource called ‘Evidence’, however, parses output files and collates relevant protein characteristics (referred henceforth as an evidence profile). A typical profile is a mixture of data types corresponding to an accuracy measure or score for the predicted characteristic (see Fig. 4). A crucial feature of the resource is a set of supervised machine learning algorithms for binary classification executed via Rscript. The ensemble of classifiers constitutes the heart of *Vacceed*’s decision making. The main output is a ranked vaccine candidate list of all proteins in the target pathogen based on an average probability of individual classifier predictions (see Fig. 4). Machine learning algorithms are the key to overcoming the challenge that an unknown percentage of evidence is questionable in each profile.

Resources encapsulate, for the most part, a large number of independent computation-intensive tasks. *Vacceed* takes advantage of multi-core processors. Part A processes one chromosome per CPU in parallel. Chromosomes are queued if there are more chromosomes than CPUs. The user, however, can specify the number of chromosomes to process in parallel. Part B internally splits the proteins by the number of CPUs and processes each subset in parallel. Alternatively, the user can specify the split value.

**Proof of concept:** There is no program yet to evaluate *in silico* vaccine candidates in a host–vaccine interaction. The best interim option is to validate the *in silico* process by predicting candidates using experimentally validated proteins with known immunogenicity characteristics i.e. compare predicted with expected to determine sensitivity and specificity of the process. Using a mixed dataset of 140 published proteins observed to induce or not induce immune responses, we demonstrated in an earlier study (Goodswen *et al.*, 2013a) *Vacceed’s* decision making potential by effectively distinguishing expected true from expected false vaccine candidates, with an average sensitivity and specificity of 0.97 and 0.98, respectively.

*Funding*: PhD scholarship from Zoetis (Pfizer) Animal Health.

*Conflict of Interest*: none declared.

## Supplementary Material

Supplementary Data
