# Supplementary material for: Vacceed: a high-throughput in silico vaccine candidate discovery pipeline for eukaryotic pathogens based on reverse vaccinology
Source: Bioinformatics. 2014 Apr 29;30(16):2381–3. doi: 10.1093/bioinformatics/btu300 (PMC4207429; doi:10.1093/bioinformatics/btu300)
Supplement: Supplementary Data [file supp_30_16_2381__index.html]

Vacceed: a high-throughput in silico vaccine candidate discovery pipeline for eukaryotic pathogens based on reverse vaccinology — Vacceed: a high-throughput in silico vaccine candidate discovery pipeline for eukaryotic pathogens based on reverse vaccinology — Vacceed: a high-throughput in silico vaccine candidate discovery pipeline for eukaryotic pathogens based on reverse vaccinology — Supplementary Data 

# *Vacceed*: a high-throughput *in silico* vaccine candidate discovery pipeline for eukaryotic pathogens based on reverse vaccinology

## Supplementary Data

files

**Files in this Data Supplement:**

- Supplementary Data - pdf file
