# Supplementary material for: Vacceed: a high-throughput in silico vaccine candidate discovery pipeline for eukaryotic pathogens based on reverse vaccinology
Source: Bioinformatics. 2014 Apr 29;30(16):2381–3. doi: 10.1093/bioinformatics/btu300 (PMC4207429; doi:10.1093/bioinformatics/btu300)
Supplement: Supplementary Data [file supp_btu300_Vacceed_User_Guide.pdf]

User Guide for *Vacceed*

Version 1.1

An *in silico* vaccine discovery pipeline

for eukaryotic pathogens

Professor John Ellis
School of Medical and Molecular Biosciences
University of Technology, Sydney (UTS)
Broadway NSW 2007, Australia
Email: John.Ellis@uts.edu.au

Vacceed – User Guide – Contents

Introduction.......... 3

Installation.......... 3

Test installation by running sample data.......... 4

How to get started.......... 5

PART A – Build Proteome.......... 7

- Quick overview of steps required prior to building proteome.......... 7
- Directory structure following *Vacceed* installation.......... 7
- Prerequisite Programs.......... 9
- Configuration of prerequisite programs.......... 11
- Prerequisite Starting Data.......... 12
- *Vacceed* Execution.......... 14
- Configuration file –startup.ini.......... 14
- Specifies configuration file.......... 15
- Resource Scripts.......... 20
- Output Files.......... 23
- Running scripts in parallel.......... 32
- Adding a new resource.......... 33

PART B – Run Pipeline.......... 36

- Quick overview of steps required prior to running pipeline.......... 36
- Directory structure following *Vacceed* installation.......... 36
- Prerequisite Programs.......... 38
- Configuration of prerequisite programs.......... 40
- Prerequisite Starting Data.......... 41
- *Vacceed* Execution.......... 41
- Configuration file –startup.ini.......... 42
- Specifies configuration file.......... 42
- Resource Scripts.......... 47
- Output Files.......... 50
- Running scripts in parallel.......... 55
- Adding a new resource.......... 56

Appendix A.......... 60

- Introduction.......... 60
- WoLF PSORT.......... 60
- SignalP.......... 62
- TargetP.......... 63

TMHMM 64
Phobius 65
MHC Binding Predictors 66
References 68

Introduction

Vacceed is the collective name for a framework of linked bioinformatics programs, Perl scripts, R functions, and Linux scripts. It has been designed to facilitate an automated, high-throughput *in silico* approach to vaccine candidate discovery for eukaryotic pathogens. This document explains how to install, configure, and run *Vacceed*. The first important point to note is not to be overwhelmed by the length of this user guide. It is not intended to be read from cover to cover but as a reference to assist in a stress-free experience in getting started and, only if desired, to obtain a more detailed understanding of how *Vacceed* works.

We recommend the following published articles for background information on motivation and theory behind *Vacceed*:

> Goodswen SJ, Kennedy PJ, Ellis JT. A guide to *in silico* vaccine discovery for eukaryotic pathogens, **Briefings in Bioinformatics** 2013;14:753-774.
> Goodswen S, Kennedy P, Ellis J. A novel strategy for classifying the output from an *in silico* vaccine discovery pipeline for eukaryotic pathogens using machine learning algorithms, **BMC Bioinformatics** 2013;14:315.

Installation

Download:

A compressed *Vacceed* directory containing all relevant Perl, R, and Linux scripts (including sample data) can be downloaded from the following URL:

https://github.com/sgoodswe/vacceed/releases

Download **vacceed_v*x.x*.tar.gz** (where *x.x* is the latest version number)

Decompress:

tar –zxvf vacceed_v*x.x*.tar.gz (where *x.x* is the latest version number)

After decompressing the downloaded *vacceed* file, the directory can be moved to any location of your choice.

Test installation by running sample data

Prior to running tests you need to install dependant programs. The programs to install depend on which of the following two main tasks you intend to perform: 1) build a proteome for the target pathogen and/or 2) run the vaccine candidate discovery pipeline. See the following section **How to get started** to assist in choosing which task to perform. Subject to your choice, refer to **Prerequisite programs** in either or both **PART A – Build Proteome** and **PART B – Run Pipeline** sections to determine which programs need to be installed. Each installed prerequisite program should be independently tested before attempting to run *Vacceed*.

Sample data for the species *Toxoplasma gondii* is provided as part of the *Vacceed* download to specifically test the installation (*T. gondii* is a eukaryotic protozoan responsible for human disease).

Test 1 – Build proteome for T. gondii

1. Edit the species configuration file 'toxoplasma_build.ini' located in the directory <*install_dir*>/vacceed/start/config_dir (where <*install_dir*> is the directory in which *Vacceed* was installed). Change the current path assigned to work_dir to the correct path to the 'vacceed' directory. Also, assign your e-mail address to email_url
2. Change directory to <*install_dir*>/vacceed/start in a command-line terminal
3. Enter the command: perl startup build tg

Note that only chromosomes 'Ia' and 'Ib' from *T. gondii* are used in the test to reduce the running time. However, step 3 can still take between 5 to 8 hours to complete depending on your computing environment. The slowness is because of one program in particular called N-Scan. It is possible to run this test in less than 10 minutes by removing N-Scan from the building process. This is achieved by removing the word NSCAN from the list of program names assigned to name under the [Resources] header in file 'toxoplasma_build.ini'. This test (including N-Scan) was completed in 6 hours: 30 minutes: 30 seconds using Red Hat Enterprise Linux Workstation release 6.4, 64 bit kernel, and 12 MB memory with 6 CPUs (without N-Scan, it only took 8 minutes).

An e-mail will automatically be sent to you either when the building of the proteome is successfully completed OR immediately when an error occurs. A log file is attached to the e-mail that provides details of the success or failure. If successful, two output files called

'proteome_info.txt' and 'proteome.fasta' are created in the directory <*install_dir*>/vacceed/toxoplasma/proteome. See **Output Files** in section **PART A – Build Proteome** for details on the contents of these files.

<u>Test 2 – run vaccine candidate discovery pipeline for T. gondii</u>

1. Edit the species configuration file 'toxoplasma.ini' located in the directory <*install_dir*>/vacceed/start/config_dir (where <*install_dir*> is the directory in which *Vacceed* was installed). Change the current path assigned to work_dir to the correct path to the 'vacceed' directory. Also, assign your e-mail address to email_url
2. Change directory to <*install_dir*>/vacceed/start in a command-line terminal.
3. Enter the command: perl startup tg

Note that only proteins encoded in chromosomes 'Ia' and 'Ib' are used in the test to reduce the running time. However, step 3 can still take between 3 to 4 hours to complete. The slowness is due to the peptide-MHC predicting. It is possible to run the test in less than 5 minutes by removing both MHCI and MHCII from the pipeline. This is achieved by removing the words MHCI and MHCII from the list of program names assigned to name under the [Resources] header in file 'toxoplasma.ini'. This test (including MHCI and MHCII) was completed in 3 hours: 21 minutes: 17 seconds using Red Hat Enterprise Linux Workstation release 6.4, 64 bit kernel, and 32 MB memory with 8 CPUs (Without MHCI and MHCII , it only took 55 seconds. MHC I took 1 hour: 25 minutes: 09 seconds and MHC II took 1 hour: 53 minutes: 51 seconds to complete).

An e-mail will automatically be sent to you either when the pipeline is successfully completed OR immediately when an error occurs. A log file is attached to the e-mail that provides details of the success or failure. If successful, two output files called 'vaccine_candidates' and 'vaccine_candidates.fasta' are created in the directory <*install_dir*>/vacceed/toxoplasma/proteome. See **Output Files** in section **PART B – Run Pipeline** for details on the contents of these files.

How to get started

The framework of *Vacceed* is organised into two major parts referred henceforth as part A – Build Proteome, and part B – Run Pipeline (see Figure below). A starting prerequisite to run the pipeline (i.e. part B) for *in silico* vaccine candidate discovery is a file in a FASTA format containing amino acid sequences for proteins from the target eukaryotic pathogen i.e. the proteome. The ultimate goal of *Vacceed* is to distinguish which of these proteins in the file are

potential vaccine candidates. Known protein sequences for many pathogens can be downloaded from public databases. Part A is therefore used only if required to predict novel protein sequences and/or collect evidence to support the existence of known proteins.

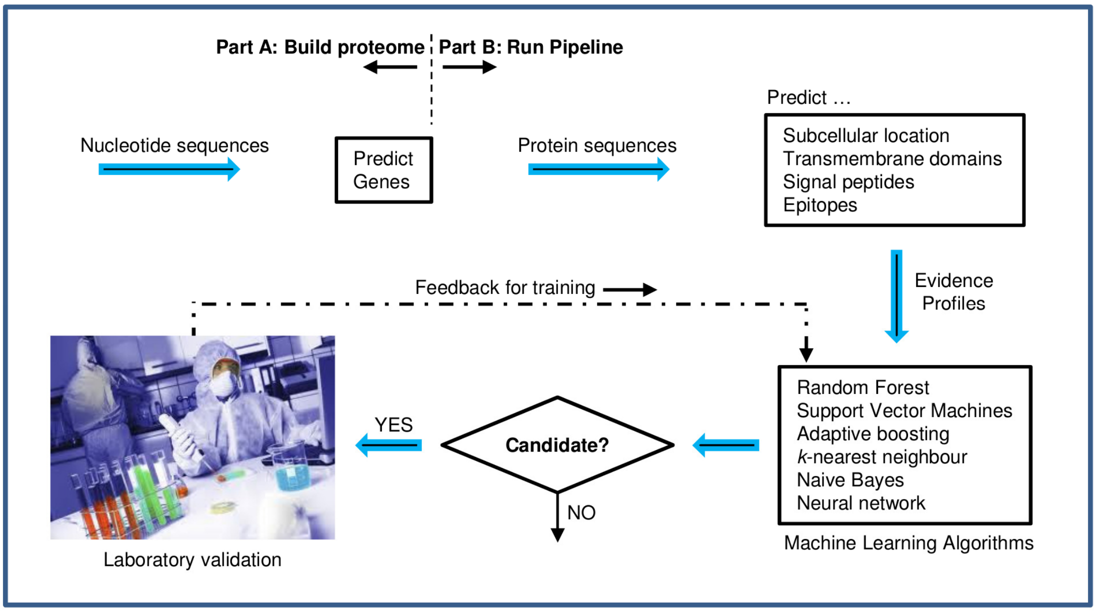

How you start to utilise *Vacceed* depends on whether you have the prerequisite file:

Starting *Vacceed* WITHOUT the prerequisite file → See section '**PART A – Build Proteome**'

Starting *Vacceed* WITH the prerequisite file → See section '**PART B – Run Pipeline**'

PART A – Build Proteome

The primary goal here is to generate a file containing the amino acid (protein) sequences in a FASTA format for all proteins from the target pathogen (referred henceforth as the proteome). The protein sequences for many pathogens can be downloaded from public databases. In light of this, the building of the proteome here entails collecting evidence to support the existence of known proteins recorded in public databases *and* the prediction of novel proteins. *Vacceed* collects evidence and makes predictions using linked resources.

Quick overview of steps required prior to building proteome

1. Install the programs Perl, BLAST, Augustus, GlimmerHMM, Blat, GMAP, and N-Scan (see section **Prerequisite Programs**).
2. Configure the installed programs, if required, for the target pathogen (see section **Configuration of prerequisite programs**).
3. Add the target pathogen to startup.ini (see section **Configuration file – startup.ini).**
4. Create a configuration file specific to the target pathogen (see **Specifies configuration file**).
5. Obtain chromosome sequences, and if available, known gene and protein sequences for the target pathogen (see **Prerequisite Starting Data**).
6. Run *Vacceed* (see **Program Execution**).

Directory structure following *Vacceed* installation

After decompressing the downloaded *Vacceed* file, the directory can be moved to any location of your choice. Figure 1 shows an example of the directory structure for the distributed version of *Vacceed*. The '?' in Figure 1 implies any pathname. Names shown in square brackets e.g. [Species Name] imply user-defined directory names. Directory names should not contain spaces e.g. Toxoplasma species is invalid, Toxoplasma_species is valid. Also, remember that Linux/UNIX is case sensitive.

The contents of the directories are:

Start – contains the Perl script to invoke *Vacceed* called startup. The master Linux script is also created in this directory.

config_dir – a directory within the start directory that contains the species specific configuration files.

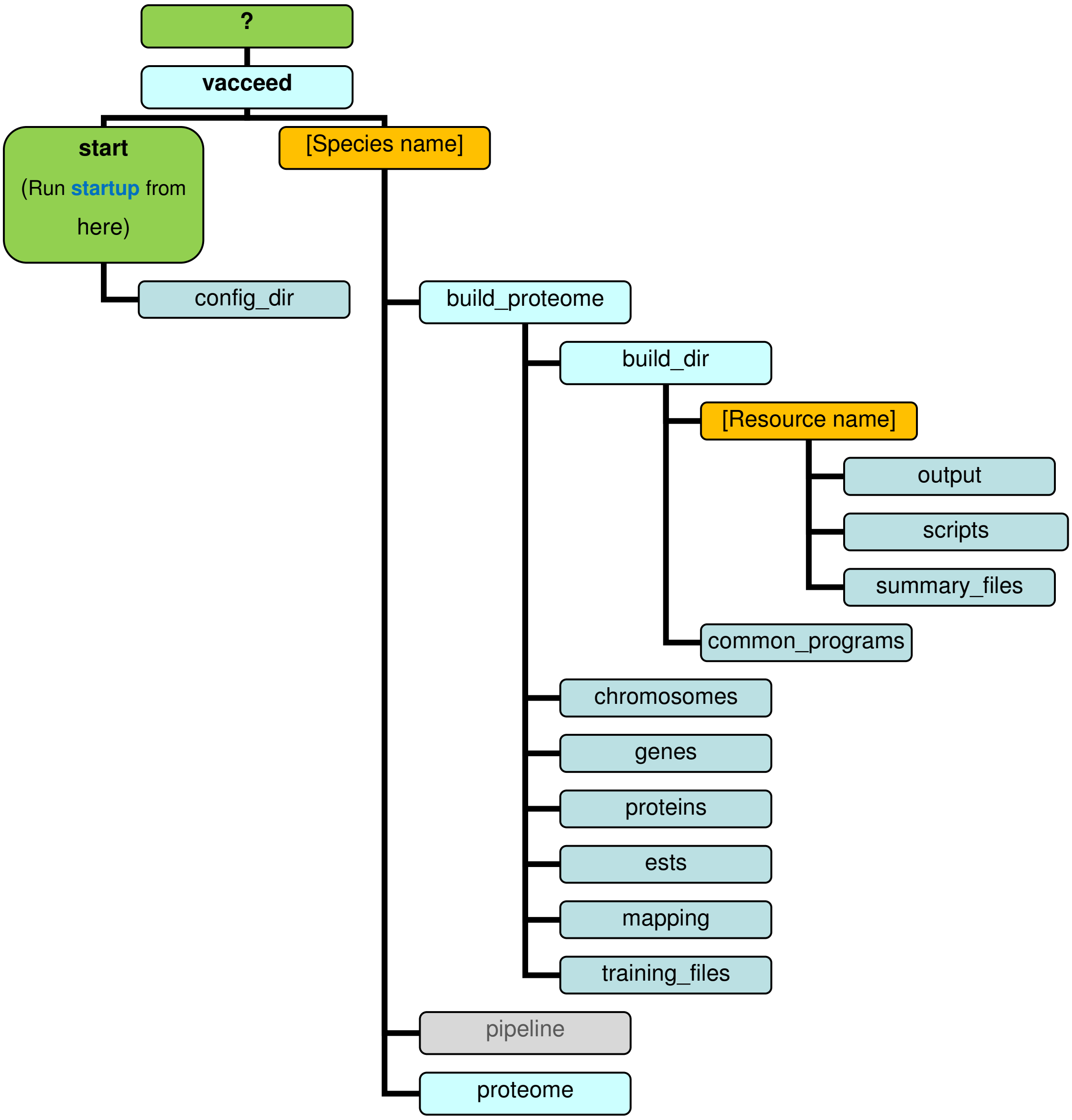

Figure 1: The *Vacceed* directory structure for the distributed version

[Species name] – A separately named but identical directory structure is used as a work area for each species. For example, the data and work area for *Toxoplasma gondii* will be in a separate user-defined directory name, perhaps called toxoplasma, and similarly for *Plasmodium falciparum* in a separate directory called plasmodium.

build_proteome – The main work area for building the proteome for the target pathogen

build_dir – contains all the resource directories used to build the proteome. Each resource has its own user named directory

[Resource name] – A separately named but identical directory structure is used for each building resource. For example, each resource directory contains three sub directories: **output** (contains the main output files from the resource programs), **scripts** (contains Linux scripts

that invoke the resource programs), and summary_files (contains summarised output files containing descriptive statistics for the genome or proteome of the target pathogen).

common_programs – contains programs that are common to more than one resource.

chromosomes – contains nucleotide sequences for each chromosome from the target pathogen. A separate file in a FASTA format is required for each chromosome. The filename should consist of a consistent prefix (e.g. chr or chromosome), a chromosome number (e.g. 1 or Ia), and a consistent extension e.g. fasta or seq. Example filenames: chr1.fasta, chr2.fasta. The FASTA sequence identifier for each chromosome should also have consistent prefix and chromosome numbering e.g >chr1|gi|401396281|, >chr2|chromosome 2|.

genes – contains nucleotide sequences for each gene within a chromosome. A separate file in a FASTA format is required for each chromosome. The filename should consist of a consistent prefix (e.g. genes_chr), a chromosome number (e.g. 1 or Ia), and a consistent extension e.g. fasta or seq. Example filenames: genes_chr1.fasta, genes_chr2.fasta.

proteins – contains amino acid sequences for all known proteins from the target pathogen. Only one file in a FASTA format is required. Any filename can be used. The FASTA sequence identifier for each protein should be in a consistent format e.g. >gi|490147168, >gi|52000749| or >tr|F0V7C2|, >tr|F0VF65|

ests – contains nucleotide sequences for Expressed Sequence Tags (ESTs). Only one file in a FASTA format is required. Any filename can be used. The FASTA sequence identifier for each EST should be in a consistent format

mapping – contains all files used to map different gene or protein identifiers e.g. map UniProt ID to NCBI gi number.

training_files – contains user created training files for resources e.g. AUGUSTUS provides the option to create a training dataset specific to the target pathogen.

proteome – contains the two most important output files. One containing resource scores for each protein and the other, the built proteome in the form of protein sequences (see section **Output Files**).

pipeline – The main work area for running the pipeline (see **Part B – Run Pipeline**).

Prerequisite Programs

Perl – *Vacceed* has been developed and tested on Perl 5.10.1 for Linux. The following Perl modules MUST be installed:

Config::Simple

File::HomeDir

The distributed version of *Vacceed* is configured to run the programs blastn, blastp, Augustus, GlimmerHMM, Blat, GMAP, and N-SCAN. These programs MUST be installed if you intend to use all resources as per the distributed version. However, you can elect to only install and/or use some of these resources by modifying a configuration file (see **Species configuration file**):

Here are the program's URLs (last viewed October 2013):
Augustus – http://bioinf.uni-greifswald.de/augustus/
GlimmerHMM – http://ccb.jhu.edu/software/glimmerhmm/
Blat – http://genome.ucsc.edu/FAQ/FAQblat.html
GMAP – http://research-pub.gene.com/gmap/
N-SCAN – http://mblab.wustl.edu/software.html (look for Twinscan/N-SCAN and the Releases link)
Blast+ –
http://blast.ncbi.nlm.nih.gov/Blast.cgi?CMD=Web&PAGE_TYPE=BlastDocs&DOC_TYPE=Download (includes blastn and blastp)

Note: Installing the prerequisite programs is perhaps the most challenging aspect to preparing *Vacceed* ready for use. It is highly recommended that you seek the help of an administrator (or an experienced Linux user). Ensure that the each program successfully runs with sample data before running *Vacceed.*

Important requirements:

[1] Append program location to the PATH variable so that the program will run from any directory. The best place to add the location is to modify the user's .bash_profile file e.g.
PATH=$PATH:$HOME/Gene_Prediction_Programs/augustus.2.5.5/bin
export PATH

[2] The name of the programs MUST be as follows (remember Linux is case sensitive):

augustus
glimmer_linux
blat
gmap
Nscan_driver.pl

Configuration of prerequisite programs

Some of the prerequisite programs need to be configured specifically for the target pathogen:

Augustus has been trained for many organisms (see the list at: http://bioinf.uni-greifswald.de/augustus/). If your target pathogen is not on the list, Augustus will need to be trained. The Augustus installation directory contains a comprehensive set of training instructions in the file 'retraining.html'. The end result of the retraining is a directory specific to the pathogen containing all relevant training files. It is recommended that you copy this directory to the training_files directory (see **Directory structure following *Vacceed* installation**); then, assign the directory name to the key 'train_file' under the AUGUSTUS resource header in the species configuration file.

GlimmerHMM has been trained on several species including *Arabidopsis thaliana*, *Coccidioides* species, *Cryptococcus neoformans*, and *Brugia malayi*, *C. elegans* and *Danio rerio* (zebrafish), and humans. If your target pathogen is not on the list, GlimmerHMM will need to be trained. Training instructions in the file 'readme.train' are located in the train directory of the GlimmerHMM installation directory. The end result of the training is a directory specific to the pathogen containing all relevant training files. It is recommended that you copy this directory to the training_files directory (see **Directory structure following *Vacceed* installation**); then, assign the directory name to the key 'train_file' under the GLIMMER resource header in the species configuration file.

GMAP requires a genome database to be created for the target pathogen. Instructions on how to do this are in the README file located in the gmap_install directory. Data for gmap genome database are typically saved in a user-defined directory under gmapdb in the gmap installation. This user-defined directory name needs to be assigned to the key 'gmap_db' under the GMAP resource header in the species configuration file. The directory path to the gmap genome database also needs to be assigned to the key 'gmap_path'

N-SCAN uses a configuration file called nscandriver.config, which is provided with the N-SCAN installation. However, a copy of nscandriver.config is also in the nscan resource directory of the *Vacceed* installation. This file needs to be modified to conform to your installation. For example, absolute paths to tmpdir, nscan, repmask (optional), blastz, lav2maf, and maf2align are required. Note that the variable $HOME is not valid in nscandriver.config. The name and location of the N-Scan configuration file needs to be assigned to the key 'nscan_config' under the NSCAN resource header in the species

configuration file. N-Scan also requires a closely related genome (referred to as the informant genome) to the target pathogen. That is, a directory that contains nucleotide sequences for each chromosome from the informant organism. A separate file in a FASTA format is required for each chromosome. The filename is expected to have a prefix of 'chr', a chromosome number (e.g. 1 or Ia), and an extension of fasta e.g: chr1.fasta.The directory name containing the informant chromosome files needs to be assigned to the key 'informant_dir' under the NSCAN resource header in the species configuration file.

Prerequisite Starting Data

The only absolute mandatory data to build the proteome are nucleotide sequences. Ideally, however, known gene and protein sequences for the target pathogen are also obtained from public databases and saved in the directories as specified below. Without these existing genes and proteins, *Vacceed* can only assume that any predicted genes and proteins are novel. Expressed sequence tags, although not mandatory, provide useful evidence to support existing or novel genes.

Nucleotide sequences for each chromosome

All the resources distributed with *Vacceed* require nucleotide sequences as input. For example, the programs Augustus and GlimmerHMM are *ab initio* gene predictors i.e. they predict genes from DNA sequence alone. Blat and GMAP align expressed sequence tags (ESTs) to DNA. A separate file in a FASTA format is required for each chromosome. The filename should consist of a consistent prefix (e.g. chr or chromosome), a chromosome number (e.g. 1 or Ia), and a consistent extension e.g. fasta or seq. Example filenames: chr1.fasta, chr2.fasta. The FASTA sequence identifier for each chromosome should also have consistent prefix and chromosome numbering e.g >chr1|gi|401396281|, >chr2|chromosome 2|. The chromosome files should be saved in the chromosome directory (see **Directory structure following *Vacceed* installation).**

Gene sequences for each chromosome

Vacceed uses resources such as Augustus and GlimmerHMM to predict genes. The predictions are compared to known genes (i.e. those recorded in public databases) using blastn. A score is determined based on the percentage of query coverage * sequence percentage similarity (see **Output Files**). The score provides evidence to support the existence of the known genes. A separate file in a FASTA format is required to contain the known genes for each chromosome. The filename should consist of a consistent prefix (e.g. genes_chr), a chromosome number (e.g. 1 or Ia), and a consistent extension e.g. fasta or seq.

Example filenames: genes_chr1.fasta, genes_chr2.fasta. The gene files should be saved in the genes directory (see **Directory structure following *Vacceed* installation).** To link the evidence for the known genes to known proteins a mapping file is required (see **Mapping requirements** below).

Protein Sequences

Predicted gene sequences by resources such as Augustus and GlimmerHMM are translated to amino acid (protein) sequences. The predicted proteins sequences are compared to known proteins (i.e. those recorded in public databases) using blastp. A score is determined based on the percentage of query coverage * sequence percentage similarity (see **Output Files**). The score provides evidence to support the existence of the known protein. Only one file in a FASTA format is required. Any filename can be used but the name used must be assigned to the key 'protein_fasta' in the species configuration file (see **Species configuration file**). The protein file should be saved in the proteins directory (see **Directory structure following *Vacceed* installation).** The FASTA sequence identifier for each protein should be in a consistent format with the same prefix e.g. 'gi' for >gi|490147168| or 'tr' for >tr|F0V7C2. *Vacceed* uses the second entity in the identifier as the protein ID e.g. 490147168 and F0V7C2 from the previous example. The default prefix is assigned to the key 'prot_id_prefix' in the species configuration file.

Expressed Sequence Tag (EST) sequences

The programs Blat and GMAP are used to align EST sequences to the chromosome sequences. Partial genes are then constructed. These predicted partial genes are compared to known genes in the genes directory using blastn. A score is determined based on the percentage of query coverage * sequence percentage similarity (see **Output Files**). The score provides evidence to support the existence of the known genes. Only one file containing the ESTs in a FASTA format is required. Any filename can be used but the name used must be assigned to the key 'est_file' in the species configuration file (see **Species configuration file**). The EST file should be saved in the ests directory (see **Directory structure following *Vacceed* installation).**

Mapping requirements

Vacceed needs to link the genes in the genes directory to the proteins in the proteins directory and vice versa. Two mandatory mapping files are required:

Gene ID to protein ID

Protein ID to Gene ID and chromosome number.

The mapping format is one link per line separated by a space or tab e.g.

#	1=Protein ID	2=Gene ID	3=Chromosome Number
	FoV7C2	NCLIV_0012	Ia

The names of the mapping files must be assigned to the appropriate keys 'map_gene_protein' and 'map_protein_gene' in the species configuration file (see **Species configuration file**). The mapping files should be saved in the mapping directory (see **Directory structure following *Vacceed* installation).** By default *Vacceed* expects a direct link between the gene and protein IDs. However, an intermediate mapping file can be used, if required, by assigning a name to the key 'map_extra'. For example, the ID for the protein sequences may be UniProt IDs but the gene IDs can only be directly linked to NCBI GI numbers. In this instance, a UniProt to GI map file should be created.

Hint: UniProt (http://www.uniprot.org/) provides a user-friendly ID mapping option.

Vacceed Execution

Vacceed is invoked with a Perl script from within a Linux\Unix shell. The Perl script is called startup and is located in the **start** directory (See Figure 1).

To **build proteome**:

Change directory to ~/vacceed/start and type…

perl startup build <pathogen> e.g. perl startup build tg

Description of arguments:

'build' instructs the script to build the proteome of the target pathogen

<pathogen> is a user-definable name that determines which configuration file to use (see **configuration file startup.ini**)

Configuration file –startup.ini

Each target pathogen requires its own configuration files (see **Specifies configuration file**). Typically there is one species configuration file for building proteome, and one for running the *in silico* vaccine discovery pipeline. An argument passed to the startup script dictates which species configuration file to use. For example, perl startup build tg or perl startup tg, where 'tg' is a user-definable code in the configuration file **startup.ini** (see Figure 2).

```
#Startup.ini – User defined configuration files
code<species<type e.g. build or pipeline<config
tg<Toxoplasma gondii<build<toxoplasma_build.ini
tg<Toxoplasma gondii<pipeline<toxoplasma.ini
```

Figure 2: startup.ini – contains a list of species configuration files.

The **startup.ini** configuration file contains 4 columns separated by a ‘<’ character. The first column can be any number of characters and is used by the startup script to make the association with the appropriate species configuration file. Column 2 is simply a description and is not used by ant program. Column 3 should be either ‘build’ to indicate that the species configuration file relates to building the proteome or ‘pipeline’ to indicate the configuration file is used to run a vaccine discovery pipeline. Column 4 is the species configuration filename (any user definable name). The startup.ini file is located in the start directory (see Figure 1).

Specifies configuration file

The core of *Vacceed* is a species configuration file in a header-key format. User-definable configuration files are required for each species. Typically, each species will have two configuration files: one for building the proteome and one for running the pipeline. Four example configuration files (template_build.ini, template.ini, toxoplasma_build.ini, toxoplasma.ini) are supplied with the *Vacceed* distribution. The distributed configuration files are found in config_dir under the start directory (see Figure 1).

The following section describes the content of a typical configuration file and presents an example of how a user can modify the contents to suit the target species. The first three steps in configuring *Vacceed* for a new target pathogen is to: 1) add new line in **startup.ini**, 2) copy template_build.ini (or toxoplasma_build.ini or another species configuration file for building a proteome if available) to <new_species_build>.ini, 3) copy the entire template_species directory to a user-named directory e.g. neospora for *Neospora caninum*.

The <new_species_build>.ini configuration file needs to be modified appropriately for the target pathogen. Any line in the configuration file that begins with ‘#’ is interpreted by the script as a comment. The *Vacceed* framework is built around the concept of a resource. A resource, in this context, is a program or group of programs executed as an independent modular unit. That is, each module contains everything necessary to execute only one aspect

of the desired functionality and can be run independently. Modular units improve maintainability and allow new resources to be added when required (see **Adding a new resource**).

The species configuration file is in header-key format (see Figure 3). For example, [Resources] is regarded as the header, and 'name' is the key. Once startup is invoked, the script executes in turn each resource listed after the 'name' key. A resource name e.g. AUGUSTUS in principle can be any name on the provision that the same name is used consistently throughout the rest of the configuration file. For example, instead of AUGUSTUS one could use 'Aug gene prediction'. The resource names can be in any order and/or excluded with the exception of ASSEMBLY, which must always be the last in the list. The distribution version of *Vacceed* uses the following programs: Augustus, GlimmerHMM, Blat, GMAP, and N-Scan as part of the process of building a proteome (See **Prerequisite Programs**)

```
# Configuration file for building Toxoplasma gondii proteome Sept 2013
[Resources]
name=AUGUSTUS,GLIMMER,BLAT,GMAP,NSCAN,ASSEMBLY
```

Figure 3: Resources – extract from a species configuration file

In most cases, only keys under the Main header (see Figure 4) will need to be modified by the user. Any key can be used as a variable replacement in the rest of the configuration file. That is, a '$' character preceding a word denotes a variable e.g. $work_dir is replaced by '$HOME/vacceed' throughout the configuration file on execution of startup.

Description of keys under Main header:

work_dir:

The path to the directory that contains the *Vacceed* installation

species_dir

The directory name that will contain all data and output for a specific species

chromosomes

A list of chromosomes to process. The chromosome number must be consistent with the number used for the chromosome filename e.g. use Ia if the filename is chrIa.fasta. The proteome is built from one chromosome at a time. That is, the execution of a resource will loop in accordance with the number of chromosomes in the list.

master_script

A Linux script file, using the name specified, will be created to collectively contain all the commands to execute each resource. This file can be modified and executed to build the proteome without the need for startup and configuration files. It is useful for debugging.

build_script_only

A 'YES' or 'NO' to indicate if only the master script should be created without building of the proteome

log_file

A directory path and a name for the log file. A log file containing program execution details (including errors if they occur) is created.

email_url

E-mail address. An e-mail will be sent either to indicate that the proteome was built successfully OR failed. A log file is attached to the e-mail.

```
[Main]
work_dir="$HOME/vacceed"
species_dir="toxoplasma"
chromosomes="Ia Ib II III"
master_script="master_script"
build_script_only="NO"
log_file="$work_dir/$species_dir_logfile.txt"
email_url=Fred.Bloggs@student.uts.edu.au
```

Figure 4: Main – extract from a species configuration file

The keys under the Variables header (see Figure 5) are essentially used to save on typing and limit the number of changes required to the configuration file. The user can add any number of variables.

```
[Variables]
protein_fasta="UniProt_proteins.fasta"
prot_id_prefix="tr"
chr_dir="$work_dir/$species_dir/build_proteome/chromosomes"
gene_dir="$work_dir/$species_dir/build_proteome/genes"
est_dir="$work_dir/$species_dir/build_proteome/ests"
prot_dir="$work_dir/$species_dir/build_proteome/proteins"
train_dir="$work_dir/$species_dir/build_proteome/training_files"
map_dir="$work_dir/$species_dir/build_proteome/mapping"
proteome_dir="$work_dir/$species_dir/proteome"
assembly_dir="$work_dir/$species_dir/build_proteome/build_dir/assembly/output"
common_dir="$work_dir/$species_dir/build_proteome/build_dir/common_programs"
map_gene_protein="map_gene_uniprot.txt"
map_protein_gene="map_uniprot_gene_chr.txt"
map_extra="map_uniprot_gi.txt"
resource_dir="[Resources.name]" # do not change
```

Figure 5: Variables – extract from a species configuration file

Description of keys under Variables header (see also section on **Directory structure following *Vacceed* installation**):

proteome_fasta = filename containing protein sequences from the target pathogen

prot_id_prefix = characters that precede the protein ID in the FASTA file specified with proteome_fasta key. For example, ‘sp’ for >sp|QQAAA| or ‘tr’ for >tr|QQBBB|. Note that the various descriptions in a FASTA definition are separated by the character ‘|’. Do not enter this character here.

chr_dir = Path to directory containing the chromosome files

gene_dir = Path to directory containing the gene files

est_dir = Path to directory containing Expressed Sequence Tags (ESTs) file

prot_dir = Path to directory containing the protein file

train_dir = Path to directory containing training files for resources

map_dir = Path to directory containing files used in mapping identifiers

assembly_dir = Path to directory that will contain the files used to build proteome

common_dir = Path to directory containing programs that are common to more than one resource.

resource_dir = value for this key must not change. This is a special case in which $resource_dir is replaced by the relevant resource name e.g. augustus.

Each resource has a possible four sections defined by 4 headers – [<resourceName >], [<resourceName >_files], [<resourceName >_programs], and [<resourceName >_arguments]. The resourceName should be consistent with the name of the resource used under the Resources header. Figure 6 represents a typical configuration for a resource. The directory for each resource contains an identical structure of three directories, which by default are called output, scripts, and summary_files.

```
[resourceName]
prog_dir="$work_dir/$species_dir/build_proteome/build_folder/$resource_dir"
script_dir="$work_dir/$species_dir/build_proteome/build_folder/$resource_dir/scripts"
out_dir="$work_dir/$species_dir/build_proteome/build_folder/$resource_dir/output"
sum_dir="$work_dir/$species_dir/build_proteome/build_folder/$resource_dir/summary_files"

[resourceName_files]
train_file="species_all"

[resourceName _programs]
1="resource_script"

[resourceName _arguments]
1="$chromosomes $chr_dir $gene_dir $train_file $out_dir $common_dir"
```

Figure 6: Resource Name – extract from a species configuration file

Description of keys under resourceName:

prog_dir

The path to the directory that contains the Linux script for the resource. Typically the resource directory.

script_dir

The directory name that will contain Linux scripts to execute the various commands of the resource. A separate script is created for each chromosome e.g. script_Ia, script_Ib. These scripts are run either in parallel or consecutively (see section on **Running scripts in parallel**). The scripts can be run independently and are useful for debugging.

out_dir

The directory name that will contain all output files generated from the resource. The output is typically divided into output data for one chromosome per file.

sum_dir

The directory name that will contain summary files. These summary files provide statistical descriptions of the output on a chromosome per chromosome basis but in one file (See section on **Output Files**)

The list of keys under the header resourceName_files is used to specify variables for filenames. These variables are used as arguments to the resource script. It is highly recommended that careful attention is made to checking filenames because they may be species-specific.

The programs to run for each resource are numerically listed under the header resourceName_programs. Only one Linux script is listed for each resource in the distribution version of *Vacceed*. This resource script typically contains all commands required to run other scripts and/or programs associated with the resource (see **Resource Scripts**). There is no limit to the number of programs that can be listed under this header. Each program is executed in numerical order commencing from key '1'.

Each program listed under resourceName_programs requires a corresponding list of arguments under the header resourceName_arguments. For example, the arguments following key '1' are associated with the program following key '1' (e.g. resource_script). It is critical the argument variables are retained in the correct order.

Resource Scripts

In the distribution version of *Vacceed*, each resource has one main Linux script (typically named after the resource) that creates a new script for each chromosome to be processed. These scripts are saved in the scripts directory (see Figure 1). Each chromosome script (e.g. scriptIa) contains all the required commands to execute the resource and to extract relevant data for that particular chromosome. The extracted data is analysed and then used to build the proteome incrementally on a chromosome by chromosome basis. There is a set hierarchal structure for the execution of all *Vacceed* scripts e.g startup → master_script → resource_script → chromosome_script. Any script can be run independently, which is ideal for debugging. Each resource script is constructed from a generic format. Figure 7 shows an example of this format. There are four main sections in the script:

Get command-line arguments – these arguments are passed by startup. The resource arguments to pass are read from the appropriate species configuration file (see section

Specifies configuration file). The arguments constitute the variables (e.g. denoted by the prefix ‘$’) used throughout the rest of the script.

# Hard coding – local variables are recommended to be added here. bg_mode is required to implement parallel processing of scripts (see section on **Running scripts in parallel).**

#Main loop for writing scripts – the idea behind this section is to create a subordinate script that encapsulates all the commands required to process and manipulate data for a given chromosome. The general pattern for each step or command to be performed is to add one line as a description of the step, and another line with the actual command. Each command line should also include ‘|| error_exit’. A generic function called error_exit is executed in the event of an error raised by the command. All errors are written to the log file. A generic script called ‘error_script’ is used to write the error_exit function.

#Run the scripts – a generic script called ‘run_scripts’ is used to execute each chromosome script.

All generic scripts are located in common_programs directory (see **Directory structure following *Vacceed* installation**).

```
#!/bin/sh
#Resource used: Example
resource=example

#Get command-line arguments
chromosomes=$1
prog_dir=$2
script_dir=$3
out_dir=$4
common_dir=$2

#Hard coding
bg_mode=1 # parallel processing: 1 = ON, 0 = OFF
filename="eg.txt"

#Main loop for writing scripts
for chr_no in  $chromosomes
do
        #Write the error function to script
        $common_dir/error_script "$chr_no" "$script_dir"

        ##Program section ##
        echo "echo Chromosome $chr_no" >> $script_dir/script$chr_no
        #Step 1
        echo "script_step=\">> executing step 1\"" >> $script_dir/script$chr_no
        echo "perl $prog_dir/example1.pl chr$chr_no $out_dir" >> $script_dir/script$chr_no \
                || error_exit
        #Step 2
        echo "script_step=\">> executing step 2\"" >> $script_dir/script$chr_no
        echo '$prog_dir/unix_script_1 "$filename" "$out_dir"' >> $script_dir/script$chr_no \
                || error_exit
done

#Run scripts
$common_dir/run_scripts "$chromosomes" "$script_dir" "$bg_mode" || exit 1
```

Figure 7: Example Resource Script

Output Files

The two most important output files are proteome_info.txt and proteome.fasta, which are saved in the **proteome** directory (see **Directory structure following *Vacceed* installation**). It is recommended that these files be examined after startup has finished and no errors were detected.

proteome_info.txt – Lists all known and predicted pathogen proteins by a descending score in a table format (see example extract below). There is one protein per row. Resource scores (i.e. evidence for protein's existence) are presented in columns e.g. aug_gene, aug_prot. Resource scores are based on: query coverage * sequence percentage similarity as determined by 'blastn' or 'blastp'. Where query coverage = percent of the query sequence (i.e. predicted gene, protein, or EST sequence) that overlaps the subject sequence (i.e. known gene or protein sequence); and percentage similarity = percent similarity between the query and subject sequences over the length of the coverage area. Resource scores are reported as a value between 0 and 1. Maximum score = 1.0 i.e. 100% query coverage and 100% sequence percentage similarity. An average score for all resources is reported in the last column and this value determines the order that the proteins appear in the list. The average score is also taken into account when the final list of vaccine candidate sequences is determined (see **PART B – Output Files**).

```
#ID,aug_gene,aug_prot,blat_gene,gl_gene,gl_prot,gmap_gene,nscan_gene,nscan_prot,Average score
S8GUS9,1.00,1.00,1.00,1.00,1.00,1.00,1.00,1.00,1.00
S8GUX4,1.00,1.00,1.00,0.93,0.92,1.00,1.00,1.00,0.98
S8GVJ8,1.00,1.00,1.00,1.00,0.84,1.00,1.00,1.00,0.98
chrla_new7,0.00,0.00,0.00,0.00,0.00,0.00,0.00,0.00,0.39
S8FCI0,0.00,0.00,0.00,0.00,0.00,0.00,0.00,0.00,0.00
```

The file 'proteome_info.txt' also lists proteins that are potentially novel. A novel protein in this context is one that matches no known protein from the target pathogen and is derived from a predicted gene that matches no known gene. A novel protein is denoted by the word 'new' as part of the ID e.g. chrIa_new7. This notation also indicates which chromosome contained the gene that encoded the novel protein. Novel proteins will always have a 0 score from each resource. However, the last column for novel proteins is a probability score rather than an average of the resource scores. The probability score (a value between 0 and 1) is derived by clustering the sequences predicted by different resources that do not match to known genes or proteins. The clustering here is based on sequence similarity using blastn or blastp. An assumption made is that a cluster containing *several* sequences has a greater

probability than a cluster containing only a *few* sequences that it represents a ‘real’ sequence (i.e. a novel sequence).

proteome.fasta – Contains the amino acid sequences in a FASTA format for all known and predicted novel proteins of the target eukaryotic pathogen. This file provides the starting prerequisite for *in silico* vaccine discovery pipeline (i.e. PART B – Run Pipeline). Note that the FASTA definition line for novel proteins contains for consistency the same characters that precede the known proteins. For example, if know proteins have ‘sp’ preceding the ID in the FASTA definition line as in >sp|QQAAA|; the novel proteins will therefore have ‘sp’ preceding the ID as in >sp|chrIa_new7|. The preceding characters are assigned to the key ‘prot_id_prefix’ in the species configuration file.

If the above files are empty or have missing or unexpected data, then the next recommended step is to review the files in the summary_files directory for each resource. These summary files typically provide an overview of the output on a chromosome by chromosome basis. If the results are not what you expect, then probing the more detailed files in the output directories may provide some clues to the source of the problem. Each resource can potentially generate many output files. The output filenames, a description of their contents, and the program that generates them is listed in the following table. In general, the files represent an audit trail for the various steps performed by each resource. For the most part, you do not normally need to be concerned with these output files unless there is a requirement to scrutinise the quality of each step.

The table uses some abbreviations and terms:

prefix – represents a consistent set of characters that precede the rest of the filename. The default is chr# (where # is the number of the chromosome). The prefix allows for the grouping and processing of files on chromosome basis

– represents a number either for a chromosome or gene

Query coverage – percent of the query sequence that overlaps the subject sequence with respect to a BLAST output

Ident – percent of similarity between the query and subject sequences over the length of the query coverage

**Program Name**	**Output file name**	**Description of contents**
**Resource = AUGUSTUS**		
augustus	prefix#.gff	Gene predictions in a General Feature Format (GFF)
getAnnoFasta.pl	prefix#.aa	Gene predictions converted to amino acid sequences
aug_genes.pl	prefix#.exons	Predicted exon locations relative to gene start (ATG)
	prefix#.seq	Predicted gene sequences (default gene identifier >g#) in a FASTA format
	prefix#_check.seq	Checks if predicted genes start with ATG and stop with either TAA, TAG, or TGA
blastn	prefix#_blastn.txt	Homology between predicted genes (prefix#.seq) and known genes of the target pathogen
e_blastn.pl	prefix#_map.txt	Reports matching genes from blastn hits
	prefix#_summary.txt	Reports perfect matching genes (E-value = 0 with 100% query coverage and ident) or partially matching genes (E-value = 0 with query coverage > 75% and < 125%)
	genome_summary.txt	Reports statistics for matching genes per chromosome (file found in **summary_files** directory)
	prefix#_aug_gene_ids.txt	Lists the existing gene IDs with matching predicted genes (file found in **assembly/output** directory)
	prefix#_aug_new_gene.fasta	Predicted gene sequences with no match to existing genes (default gene identifier >Resource Name-g#) (file found in **assembly/output** directory)
blastp	prefix#_blastp.txt	Homology between predicted proteins (prefix#.aa) and known proteins of the target pathogen
e_blastp.pl	prefix#_prot_map.txt	Reports matching proteins from blastp hits
	prefix#_prot_summary.txt	Reports perfect matching proteins (E-value = 0 with 100% query coverage and ident) or partially matching proteins (default: E-value = 0 with query coverage > 75% and <

		125%)
	protein_summary.txt	Reports statistics for matching proteins per chromosome (file found in **summary_files** directory)
	prefix#_aug_prot_ids.txt	Lists the existing protein UniProt IDs with matching predicted proteins (file found in **assembly/output** directory)
	prefix#_aug_new_prot.fasta	Predicted protein sequences with no match to existing proteins (default protein identifier >Resource Name-g#) (file found in **assembly/output** directory)
blastp	prefix#_sim_blastp.txt	Homology between predicted proteins with no match to existing pathogen proteins (prefix#_aug_new_prot.fasta) and NCBI nr redundant database. Note that this step is only performed if variable blastp_on_nr is set to 1 in the augustus_script [default is 0]. It can take several days to run
e_blastp_sim.pl	prefix#_sim_summary.txt	Reports perfect matching proteins (E-value = 0 with 100% query coverage and ident) or partially matching proteins (default: E-value = 0 with query coverage > 25% and < 175%, and ident > 25%)
	prefix#_aug_sim_ids.txt	Lists the existing protein UniProt IDs with matching homologues (file found in **assembly/output** directory)
	prefix#_aug_new_sim.fasta	Predicted protein sequences with no match to any existing proteins (default protein identifier >Resource Name-g#) (file found in **assembly/output** directory)
**Resource = GLIMMER**		
glimmerhmm_linux.pl	prefix#.gff	Gene predictions in a General Feature Format (GFF)
gl_genes.pl	prefix#.exons	Predicted exon locations relative to gene start (ATG)
	prefix#.seq	Predicted gene sequences (default gene identifier >g#) in a FASTA format
	prefix#_check.seq	Checks if predicted genes start with ATG and stop with either TAA, TAG, or TGA

	prefix#_gene_start_end.txt	Predicted gene location relative to the start of the chromosome
	prefix#_mrna.seq	Predicted mRNA sequence
blastn	prefix#_blastn.txt	Homology between predicted genes (prefix#.seq) and known genes of the target pathogen
e_blastn.pl	prefix#_map.txt	Reports matching genes from blastn hits
	prefix#_summary.txt	Reports perfect matching genes (E-value = 0 with 100% query coverage and ident) or partially matching genes (default: E-value = 0 with query coverage > 75% and < 125%)
	genome_summary.txt	Reports statistics for matching genes per chromosome (file found in **summary_files** directory)
	prefix#_gl_gene_ids.txt	Lists the existing gene IDs with matching predicted genes (file found in **assembly/output** directory)
	prefix#_gl_new_gene.fasta	Predicted gene sequences with no match to existing genes (default gene identifier >Resource Name-g#) (file found in **assembly/output** directory)
convert_dna_to_aa.pl	prefix#.aa	mRNA predictions (prefix#_mrna.seq) converted to amino acid sequences
blastp	prefix#_blastp.txt	Homology between predicted proteins (prefix#.aa) and known proteins of the target pathogen
e_blastp.pl	prefix#_prot_map.txt	Reports matching proteins from blastp hits
	prefix#_prot_summary.txt	Reports perfect matching proteins (E-value = 0 with 100% query coverage and ident) or partially matching proteins (default: E-value = 0 with query coverage > 75% and < 125%)
	protein_summary.txt	Reports statistics for matching proteins per chromosome (file found in **summary_files** directory)
	prefix#_gl_prot_ids.txt	Lists the existing protein UniProt IDs with matching predicted proteins (file found in **assembly/output** directory)
	prefix#_gl_new_prot.fasta	Predicted protein sequences with no match

		to existing pathogen proteins (default protein identifier >Resource Name-g#) (file found in **assembly/output** directory)
blastp	prefix#_sim_blastp.txt	Homology between predicted proteins with no match to existing pathogen proteins (prefix#_gl_new_prot.fasta) and NCBI nr redundant database. Note that this step is only performed if variable blastp_on_nr is set to 1 in the glimmer_script [default is 0]. It can take several days to run
e_blastp_sim.pl	prefix#_sim_summary.txt	Reports perfect matching proteins (E-value = 0 with 100% query coverage and ident) or partially matching proteins (default: E-value = 0 with query coverage > 25% and < 175%, and ident > 25%)
	prefix#_gl_sim_ids.txt	Lists the existing protein UniProt IDs with matching homologues (file found in **assembly/output** directory)
	prefix#_gl_new_sim.fasta	Predicted protein sequences with no match to any existing proteins (default protein identifier >Resource Name-g#) (file found in **assembly/output** directory)
**Resource = BLAT**		
blat	prefix#_est.psl	Alignment of Expressed Sequence Tags (ESTs) with chromosome
psl_to_fasta.pl	prefix#.seq	Predicted 'partial' gene sequences derived from aligned ESTs (default gene identifier >gene_#)
blastn	prefix#_blastn.txt	Homology between predicted partial genes (prefix#.seq) and known genes of the target pathogen
e_blastn.pl	prefix#_map.txt	Reports matching genes from blastn hits
	prefix#_summary.txt	Reports perfect matching genes (E-value = 0 with 100% query coverage and ident) or partially matching genes (default: E-value = 0 with query coverage > 75% and < 125%)
	genome_summary.txt	Reports statistics for matching genes per chromosome (file found in **summary_files** directory)

	prefix#_blat_gene_ids.txt	Lists the existing gene IDs with matching predicted genes (file found in **assembly/output** directory)
	prefix#_blat_new_gene.fasta	Predicted gene sequences with no match to existing genes (default gene identifier >Resource Name-gene_#) (file found in **assembly/output** directory)
	**Resource = GMAP**	
gmap	Est_all.psl	Alignment of Expressed Sequence Tags (ESTs) with ALL chromosomes
split_psl.pl	prefix#_est.psl	Alignment of Expressed Sequence Tags (ESTs) with chromosome
psl_to_fasta.pl	prefix#.seq	Predicted 'partial' gene sequences derived from aligned ESTs (default gene identifier >gene_#)
blastn	prefix#_blastn.txt	Homology between predicted partial genes (prefix#.seq) and known genes of the target pathogen
e_blastn.pl	prefix#_map.txt	Reports matching genes from blastn hits
	prefix#_summary.txt	Reports perfect matching genes (E-value = 0 with 100% query coverage and ident) or partially matching genes (default: E-value = 0 with query coverage > 75% and < 125%)
	genome_summary.txt	Reports statistics for matching genes per chromosome (file found in **summary_files** directory)
	prefix#_gmap_gene_ids.txt	Lists the existing gene IDs with matching predicted genes (file found in **assembly/output** directory)
	prefix#_gmap_new_gene.fasta	Predicted gene sequences with no match to existing genes (default gene identifier >Resource Name-gene_#) (file found in **assembly/output** directory)
	**Resource = NSCAN**	
run_nscan.pl	prefix#.fasta.masked.gtf	Gene predictions in a Gene Transfer Format (GTF)
nscan_genes.pl	prefix#.seq	Predicted gene sequences (default gene identifier >gene_#)
	prefix#_check.seq	Checks if predicted genes start with ATG

		and stop with either TAA, TAG, or TGA
	prefix#_mrna.seq	Predicted mRNA sequence
blastn	prefix#_blastn.txt	Homology between predicted genes (prefix#.seq) and known genes of the target pathogen
e_blastn.pl	prefix#_map.txt	Reports matching genes from blastn hits
	prefix#_summary.txt	Reports perfect matching genes (E-value = 0 with 100% query coverage and ident) or partially matching genes (default: E-value = 0 with query coverage > 75% and < 125%)
	genome_summary.txt	Reports statistics for matching genes per chromosome (file found in **summary_files** directory)
	prefix#_gl_gene_ids.txt	Lists the existing gene IDs with matching predicted genes (file found in **assembly/output** directory)
	prefix#_gl_new_gene.fasta	Predicted gene sequences with no match to existing genes (default gene identifier >Resource Name-g#) (file found in **assembly/output** directory)
Convert_dna_to_aa.pl	prefix#.aa	mRNA predictions (prefix#_mrna.seq) converted to amino acid sequences
blastp	prefix#_blastp.txt	Homology between predicted proteins (prefix#.aa) and known proteins of the target pathogen
e_blastp.pl	prefix#_prot_map.txt	Reports matching proteins from blastp hits
	prefix#_prot_summary.txt	Reports perfect matching proteins (E-value = 0 with 100% query coverage and ident) or partially matching proteins (default: E-value = 0 with query coverage > 75% and < 125%)
	protein_summary.txt	Reports statistics for matching proteins per chromosome (file found in **summary_files** directory)
	prefix#_gl_prot_ids.txt	Lists the existing protein UniProt IDs with matching predicted proteins (file found in **assembly/output** directory)
	prefix#_gl_new_prot.fasta	Predicted protein sequences with no match to existing pathogen proteins (default protein

		identifier >Resource Name-g#) (file found in **assembly/output** directory)
blastp	prefix#_sim_blastp.txt	Homology between predicted proteins with no match to existing pathogen proteins (prefix#_gl_new_prot.fasta) and NCBI nr redundant database. Note that this step is only performed if variable blastp_on_nr is set to 1 in the glimmer_script [default is 0]. It can take several days to run
e_blastp_sim.pl	prefix#_sim_summary.txt	Reports perfect matching proteins (E-value = 0 with 100% query coverage and ident) or partially matching proteins (E-value = 0 with query coverage > 25% and < 175%, and ident > 25%)
	prefix#_gl_sim_ids.txt	Lists the existing protein UniProt IDs with matching homologues (file found in **assembly/output** directory)
	prefix#_gl_new_sim.fasta	Predicted protein sequences with no match to any existing proteins (default protein identifier >Resource Name-g#) (file found in **assembly/output** directory)
**Resource = ASSEMBLY**		
merge_fasta.pl	prefix#_merged_gene.fasta	Merged resource predicted gene sequences with no match to existing genes .i.e. prefix#_<*resource name*>_new_gene.fasta files are combined (default gene identifier >Resource Name-g#)
	prefix#_merged_protein.fasta	Merged resource predicted protein sequences with no match to existing genes .i.e. prefix#_<*resource name*>_new_prot.fasta files are combined (default gene identifier >Resource Name-g#)
blastn	prefix#_merged_blastn.txt	Homology between predicted genes in prefix#_merged_gene.fasta
e_blastn_new.pl	prefix#_scored_genes.txt	Reports clusters of predicted genes that match with blastn hits (default ident and coverage thresholds are 95% and 75% respectively). The number of unique

		members in the cluster is used as a score. Clusters are listed in descending score.
blastp	prefix#_merged_blastp.txt	Homology between predicted genes in prefix#_merged_protein.fasta
e_blastp_new.pl	prefix#_scored_proteins.txt	Reports clusters of predicted proteins that match with blastp hits (default ident and coverage thresholds are 95% and 75% respectively). The number of unique members in the cluster is used as a score (scores from prefix#_scored_genes.txt are included). Clusters are listed by descending score.
	new_prot.fasta	Proteins sequences for protein clusters. Sequence derived from the member with the longest sequence. These sequences are potential novel proteins. Default protein identifier = >chr#_new# (file found in **proteome** directory)
assemble.pl	proteome_info.txt	Lists all know proteins by descending score in a table format. There is one protein per row. Resource scores (i.e. evidence for protein's existence) are presented in columns. The total score (shown in last column) for each protein is derived from the addition of the individual resource scores. Resource scores are based on (query coverage * ident). Maximum resource score = 1.0
build_proteome.pl	proteome.fasta	Amino acid sequences in FASTA format for all known and novel proteins i.e. merged known proteins FASTA file with new_prot.fasta

Running scripts in parallel

The resources encapsulate, for the most part, a large number of independent computation-intensive tasks. *Vacceed* takes advantage of multi-core processors. The default when building the proteome is to processes one chromosome per CPU in parallel. Chromosomes are queued it there are more chromosomes than CPUs i.e. when a chromosome has finished processing a

new one will commence. The user can specify the number of chromosomes to process in parallel by altering the number assigned to the variable 'no_in_parallel' in run_scripts located in the common_programs directory. The chromosomes can be processed consecutively if setting for bg_mode = 0 (default is bg_mode=1 for parallel processing – see **Resource Scripts**).

Adding a new resource

This section describes the steps required to add a new resource. It is assumed here that the resource is to contain a fictitious program called program_x that predicts genes from chromosome sequences. The primary goal is to deduce protein sequences from the gene sequences:

1. Install program_x and append program location to the PATH variable so that the program will run from any directory. The best place to add the location is to modify the user's .bash_profile file.
   E.g. *PATH=$PATH:$HOME/Gene_Prediction_Programs/program_x/bin*

2. Test that the program will run with sample data and ensure it can be invoked from any directory. Furthermore, determine the input and output requirements.

3. Add a new resource name in the appropriate configuration file for building the proteome

   ```
   [Resources]
   name=AUGUSTUS,GLIMMER,BLAT,GMAP,NEW_RESOURCE,ASSEMBLY
   ```

4. Add a new section to the same configuration file. The easiest way to do this is to copy an existing resource and amend accordingly. The texts highlighted in red are the only parts expected to be changed (see **Species configuration file** for more details).

   ```
   [NEW_RESOURCE] ← This must be the same name as that used in step 3.
   prog_dir="$work_dir/$species_dir/build_proteome/build_folder/$resource_dir"
   script_dir="$work_dir/$species_dir/build_proteome/build_folder/$resource_dir/scripts"
   out_dir="$work_dir/$species_dir/build_proteome/build_folder/$resource_dir/output"
   sum_dir="$work_dir/$species_dir/build_proteome/build_folder/$resource_dir/summary_files"

   [NEW_RESOURCE_files]
   train_file="species_all" ← A training file may not be required for some programs
   additional_file="file.txt" ← Only if required
   ```

```
[NEW_RESOURCE_programs]
1="new_resource_script"

[NEW_RESOURCE_arguments]
1="$chromosomes $chr_dir $gene_dir $train_file $out_dir $common_dir $additional_file"
```

5. Create a new directory in the ‘build_dir’ (see **Directory structure following *Vacceed* installation)** using the same name (but in lowercase) as the new resource; then create three directories called ‘output’, ‘scripts’, and summary_files in this newly created directory.

6. Copy the ‘template_resource_script’ from the common_programs directory into the new resource directory and rename it to the same name as that specified for the program under [NEW_RESOURCES_programs] header.

7. Amend the new_resource_script from step 6 (see **Resource Scripts** for example script). Add the new program or programs within the ‘Main loop for writing scripts’ where is states “<< Add new programs here >>”. You need to ensure that the required arguments (i.e. the ‘inputs’ as determined in step 2) are passed to program_x and the output is to $out_dir (see example below).

```
#Step description
echo "script_step=\">> executing program_x\"" >> $script_dir/script$chr_no
echo "program_x $required_input $out_dir" >> $script_dir/script$chr_no \
|| error_exit
```

8. The next step is totally dependent on the output generated by program_x. Most gene prediction programs output the predictions in either a General Feature Format (GFF) or a Gene Transfer Format (GTF). You need to convert the output from program_x into gene sequences in a FASTA format. For GFF and GTF there are open source programs you can download. Alternately, for GFF you can use ‘aug_genes.pl’ from the augustus resource (or ‘gl_genes.pl’ from glimmer resource) as a template to write your own conversion Perl script. Similarly, for GTF you can use ‘nscan_genes.pl’ from the nscan resource. The conversion program and appropriate arguments need to be added to new_resource_script. The pathogen genome is processed on a chromosome per chromosome basis. The names used for the files containing gene sequences should be consistent. The default name used in new_resource_script is

‘chr#.seq’, where # is the chromosome number. Also the gene identifiers e.g. >gene_# should be consistent, where # in this case is a consecutive gene count.

Example

```
echo "script_step=\"conversion_program.pl for $chr_no\"" >> $script_dir/script$chr_no
echo "perl $prog_dir/conversion_program.pl chr$chr_no $chr_dir $out_dir \
|| error_exit" >> $script_dir/script$chr_no
```

9. The next step is also totally dependent on the output generated by program_x. Some gene prediction programs automatically convert gene sequences to protein sequences and some do not. In the latter case, you will need to find a program to do the conversion. Note that exon locations must be taken into account to obtain the mRNA sequence before the DNA to amino acid codon translation. Perl scripts such as ‘getAnnoFasta.pl’ from augustus resource or ‘convert_dna_to_aa.pl’ from glimmer resource (converts mRNA to amino acid sequences) could be used as templates for your own conversion program. The conversion program and appropriate arguments need to be added to new_resource_script. The names used for the files containing protein sequences should be consistent. The default name used in new_resource_script is ‘chr#.aa’, where # is the chromosome number. Also the protein identifiers e.g. >gene_# should be consistent with the gene identifiers.

```
echo "script_step=\"conversion_dna_to_aa.pl for $chr_no\"" >> $script_dir/script$chr_no
echo "perl $prog_dir/conversion_dna_to_aa.pl chr$chr_no $chr_dir $out_dir \
|| error_exit" >> $script_dir/script$chr_no
```

10. Check the rest of the commands in the new_resource_script to ensure that they are appropriate. These commands will compare the predicted gene and protein sequences with existing ones using blastn and blastp respectively; determine homology between predicted proteins with no match to existing pathogen proteins (only if variable blastp_on_nr is set to 1); create gene and protein summary information per chromosome. The commands will also collect the appropriate data for the assembly resource to build the proteome.

    Provided you have added relevant programs to create files containing gene and protein sequences, no further amendments or steps are required.

PART B – Run Pipeline

This section describes everything you need to know to automate the process of high-throughput *in silico* vaccine candidate discovery for eukaryotic pathogens. The primary goal here is to computationally generate a file containing only the protein sequences (in a FASTA format) that represent the predicted vaccine candidates for the target pathogen. A prerequisite to run the pipeline is a file in a FASTA format containing protein sequences from the target pathogen. These protein sequences can be downloaded from public databases and/or predicted following the steps described in **Part A – Build Proteome**.

The pipeline in this context is a framework of data-processing stages. Each stage in the pipeline is encapsulated as a resource that mainly contains commands to execute a central bioinformatics program and pre and post-processing auxiliary scripts/programs. A design objective of the pipeline was to have a seamless transition from input to final output. The transition is achieved by internal Perl scripts. The central bioinformatics programs, in the distributed version of *Vacceed*, are freely available programs used to predict protein characteristics (i.e. potential evidence). These programs and their typical outputs are described in **Appendix A**. An internal resource called 'EVIDENCE' collects all the evidence and performs a binary classification of the input proteins using a pool of machine learning algorithms.

Quick overview of steps required prior to running pipeline

1. Install the programs Perl, WoLF PSORT, SignalP, TargetP, TMHMM, Phobius, and IEDB peptide-MHC binding predictors (see section **Prerequisite Programs**).
2. Configure the installed programs, if required, for the target pathogen (see section **Configuration of prerequisite programs**).
3. Add the target pathogen to startup.ini (see section **Configuration file –startup.ini).**
4. Create a configuration file for the target pathogen (see **Specifies configuration file**).
5. Run *Vacceed* (see **Program Execution**).

Directory structure following *Vacceed* installation

Figure 8 shows an example of the directory structure for the distributed version of *Vacceed.* The '?' in Figure 8 implies any pathname. Names shown in square brackets e.g. [Species Name] imply user-defined directory names. Directory names should not contain spaces e.g.

Toxoplasma species is invalid, Toxoplasma_species is valid. Also, remember that Linux/UNIX is case sensitive.

The contents of the directories are:

Start – contains the Perl script to invoke *Vacceed* called startup. The master Linux script also is created in this directory.

config_dir – a directory within the start directory that contains the species specific configuration files.

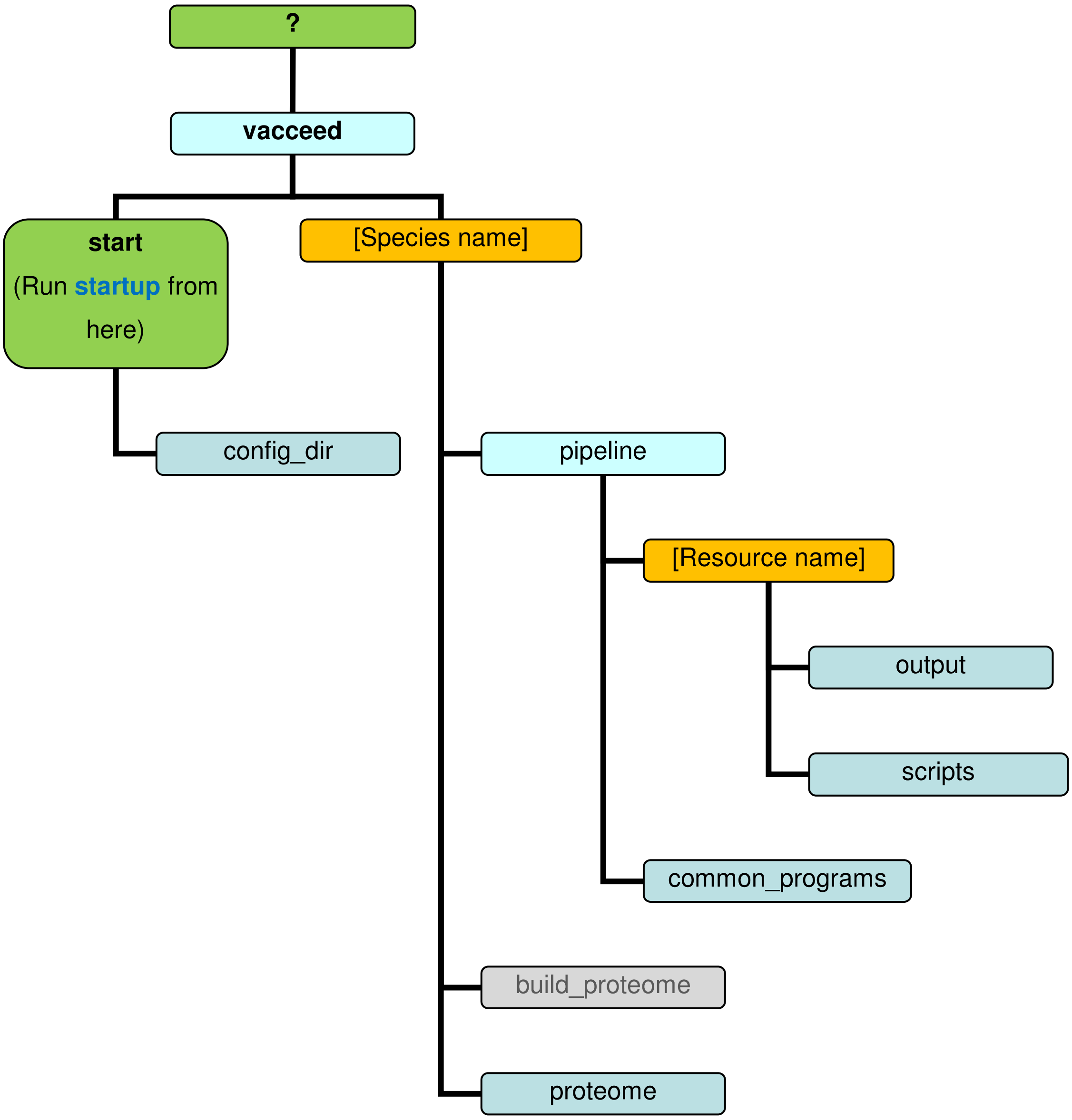

Figure 8: The *Vacceed* directory structure for the distributed version

[Species name] – A separately named but identical directory structure is used as a work area for each species. For example, the data and work area for *Toxoplasma gondii* will be in a

separate user-defined directory name, perhaps called toxoplasma, and similarly for *Plasmodium falciparum* in a separate directory called plasmodium.

pipeline – The parent directory that contains all the resource directories.

[Resource name] – A separately named but identical directory structure is used for each evidence prediction resource. For example, each resource directory contains two sub directories: **output** (contains the main output files from the resource programs), **scripts** (contains Linux scripts that invoke the resource programs). Some resources contain an additional directory called training_files, which contain the necessary file for training the resource.

common_programs – contains programs that are common to more than one resource.

proteome – contains the prerequisite input file to run the pipeline i.e. a file containing protein sequences from the target pathogen in a FASTA format. Also, contains the main output file from the pipeline, which is a file containing protein sequences for predicted vaccine candidates in a FASTA format.

Prerequisite Programs

Perl – *Vacceed* has been developed and tested on Perl 5.10.1 for Linux. The following Perl modules MUST be installed:

Config::Simple
File::HomeDir

The distributed version of *Vacceed* is configured to run the programs WoLF PSORT, SignalP, TargetP, TMHMM, Phobius, and IEDB peptide-MHC binding predictors. These programs MUST be installed if you intend to use all resources as per the distributed version. However, you can elect to only install some of the resources and then modify the list of resources to use via the key 'name' under the [Resources] header in the species configuration file.

Here are the program's URLs (last viewed October 2013):

WoLF PSORT (0.2) – http://wolfpsort.seq.cbrc.jp/

SignalP (v4.1) – http://www.cbs.dtu.dk/services/SignalP/

TargetP (v1.1) – http://www.cbs.dtu.dk/services/TargetP/

TMHMM (v2.0) – http://www.cbs.dtu.dk/services/TMHMM/

Phobius (1.01) – http://phobius.sbc.su.se/

MHC I binding predictor (v2.9.1) – http://tools.immuneepitope.org/mhci/download/

MHC II binding predictor (v2.5.1) – http://tools.immuneepitope.org/mhcii/

Note: Installing the prerequisite programs is perhaps the most challenging aspect to preparing *Vacceed* ready for use. It is highly recommended that you seek the help of an administrator (or an experienced Linux user). Ensure that the each program successfully runs with sample data before running *Vacceed.*

Important requirements:

[1] Append program location to the PATH variable so that the program will run from any directory. The best place to add the location is to modify the user's .bash_profile file e.g.

PATH=$PATH:$HOME/pipeline_programs/signalp

export PATH

[2] *Vacceed* uses the default program names in the equivalent resource scripts. The expected default names of the programs are as follows (remember Linux is case sensitive):

WoLF PSORT – runWolfPsortSummary

SignalP – signalp

TargetP – targetp

TMHMM – tmhmm

Phobius – phobius.pl

MHC I binding predictor – predict_binding.py

MHC II binding predictor – mhc_II_binding.py

Each of the above programs is referred to a as a resource in *Vacceed*. Consequently each program, as do all resources, has a specific Linux script that contains all relevant commands to execute the program. For the most part, these scripts will not need to be altered unless a program parameter is required to be changed.

[3] The distributed version of *Vacceed* is configured to run machine learning algorithms via R functions contained in packages. The following R packages MUST be installed if you intend to use all algorithms as per the distributed version. However, you can elect to only install some of the packages and then modify the list of algorithms to use via the key 'algorithms' under the [EVIDENCE] header in the species configuration file.

Machine learning algorithm and associated R packages:

Adaptive boosting– *ada*

Random forest – *randomForest*

***k*-nearest neighbour classifier** (*k*-NN) – *knn* R function contained in *Class* package

Naive Bayes classifier – *naiveBayes R* function contained in the *e1071* package

Neural network – *nnet* R function contained in the *nnet* package

Support vector machines (SVM) – *ksvm* R function contained in *kernlab* package.

The algorithms are executed using Rscript. There are three R functions that encapsulate the relevant command for each algorithm: <*al*>_wrapper.R, <*al*>_runPred.R, and <*al*>_makePred.R; where <*al*> is an algorithm abbreviation. The abbreviations used are: ada = Adaptive boosting, knn = k-nearest neighbour classifier, nb = Naive Bayes classifier, nn = Neural network, rf = Random forest, and svm = Support vector machines. The parameters to fine tune the algorithms can be modified in <*al*>_makePred.R.

Note: The predictors for the machine learning algorithms are extracted from the column headers of the 'evidence_profiles'. The EVIDENCE key called ignore_predictors is used to list the headers to ignore as predictors.

Configuration of prerequisite programs

Some of the prerequisite programs need to be configured specifically for the target pathogen:

MHC I binding predictor program needs a file containing MHC Class I alleles of the host of the pathogen. This is required for the peptide-MHC binding predictions. The file should be in a comma delimited format with columns allele name and peptide length.

Example of an allele file for cattle

```
BoLA-T2C,10
BoLA-T2C,11
BoLA-T2C,12
BoLA-T2C,13
BoLA-T2C,14
BoLA-T2C,8
BoLA-T2C,9
```

It is recommended that you copy the appropriate allele file to the 'alleles' directory under the 'mhci' parent directory; then, assign the allele filename to the key 'allele_file' under the [MHCI] resource header in the species configuration file.

MHC II binding predictor program needs a file containing MHC Class II alleles of the host of the pathogen. This is required for the peptide-MHC binding predictions. The file should have only one column containing the allele name

Example of an allele file for humans

```
H2-IAb
H2-IAd
H2-IAs
H2-IEd
HLA-DPA1*01/DPB1*04:01
HLA-DPA1*01:03/DPB1*02:01
```

It is recommended that you copy the appropriate allele file to the 'alleles' directory under the 'mhcii' parent directory; then, assign the allele filename to the key 'allele_file' under the [MHCII] resource header in the species configuration file.

WoLF PSORT has a default sequence length restriction of 10000. If your input protein sequences are likely to be greater than this restriction, it recommended that the Perl script 'checkFastaInput.pl' located in the bin directory of the WoLF PSORT installation be edited. Change the value assigned to '$maxSeqLen' e.g. my $maxSeqLen = 50000.

Prerequisite Starting Data

The only absolute mandatory input required to run the pipeline is a file in a FASTA format containing amino acid sequences for proteins from the target eukaryotic pathogen. This file must be contained in the 'proteome' directory (see **Directory structure following *Vacceed* installation).**

Vacceed Execution

Vacceed is invoked with a Perl script from within a Linux\Unix shell. The Perl script is called startup and is located in the **start** directory (See Figure 8).

To **run pipeline**:

Change directory to ~/vacceed/start and type

perl startup <pathogen>   e.g. perl startup tg

Description of arguments:

<pathogen> is a user-definable name that determines which configuration file to use (see **configuration file startup.ini**)

Configuration file –startup.ini

Each target pathogen requires its own configuration files (see **Specifies configuration file**). Typically there is one species configuration file for building proteome, and one for running the *in silico* vaccine discovery pipeline. An argument passed to the startup script dictates which species configuration file to use. For example, perl startup build tg or perl startup tg, where ‘tg’ is a user-definable code in the configuration file **startup.ini** (see Figure 9).

```
#Startup.ini – User defined configuration files
code<species<type e.g. build or pipeline<config<config directory path
tg<Toxoplasma gondii<build<toxoplasma_build.ini<$HOME/Vacceed/start/config_dir
tg<Toxoplasma gondii<pipeline<toxoplasma.ini<<$HOME/Vacceed/start/config_dir
```

Figure 9: startup.ini – contains a list of species configuration files.

The **startup.ini** configuration file contains 4 columns separated by a ‘<’ character. The first column can be any number of characters and is used by the startup script to make the association with the appropriate species configuration file. Column 2 is simply a description and is not used by ant program. Column 3 should be either ‘build’ to indicate that the species configuration file relates to building the proteome or ‘pipeline’ to indicate the configuration file is used to run a vaccine discovery pipeline. Column 4 is the species configuration filename (any user definable name). The startup.ini file is located in the start directory (see Figure 8).

Specifies configuration file

The core of *Vacceed* is a species configuration file in a header-key format. User-definable configuration files are required for each species. Typically, each species will have two configuration files: one for building the proteome and one for running the pipeline. Four example configuration files (template_build.ini, template.ini, toxoplasma_build.ini, toxoplasma.ini) are supplied with the *Vacceed* distribution. The distributed configuration files are found in config_dir under the start directory (see Figure 8).

The following section describes the content of a configuration file and presents an example of how a user can modify the contents to suit the target species. The first three steps in configuring *Vacceed* for a new target pathogen is to: 1) add new line in **startup.ini**, 2) copy

toxoplasma.ini (or another species configuration file if available) to <new_species >.ini, 3) copy the entire template_species directory to a user-named directory e.g. neospora for *Neospora caninum.*

The <new_species >.ini configuration file needs to be modified appropriately for the target pathogen. Any line in the configuration file that begins with '#' is interpreted by the script as a comment. The *Vacceed* framework is built around the concept of a resource. A resource, in this context, is a program or group of programs executed as an independent modular unit. That is, each module contains everything necessary to execute only one aspect of the desired functionality and can be run independently. Modular units improve maintainability and allow new resources to be added when required (see **Adding a new resource**).

The species configuration file is in header-key format (see Figure 9). For example, [Resources] is regarded as the header, and 'name' is the key. Once startup is invoked, the script executes in turn each resource listed after the 'name' key. A resource name e.g. WOLF in principle can be any name on the provision that the same name is used consistently throughout the rest of the configuration file. For example, instead of WOLF one could use 'WoLF_PSORT'. The resource names can be in any order with the exception of VALIDATE and EVIDENCE, which must always be the first and last in the list respectively.

```
# Configuration file for Toxoplasma gondii pipeline Sept 2013
[Resources]
name=VALIDATE,WOLF,TMHMM,SIGNALP,TARGETP,PHOBIUS,MHCI,MHCII,EVIDENCE
```

Figure 9: Resources – extract from a species configuration file

The distribution version of *Vacceed* uses the following programs: WoLF PSORT, SignalP, TargetP, TMHMM, Phobius, and IEDB peptide-MHC binding predictors to predict various protein characteristics (see section **Prerequisite Programs**). These programs represent the prediction resources.

In most cases, only keys under the Main header (see Figure 10) will need to be modified by the user. Any key can be used as a variable replacement in the rest of the configuration file. That is, a '$' character preceding a word denotes a variable e.g. $work_dir is replaced by '$HOME/vacceed' throughout the configuration file on execution of startup.

Description of keys under Main header:
work_dir:

The path to the directory that contains the *Vacceed* installation

species_dir

The directory name that will contain all data and output for a specific species

master_script

A Linux script file, using the name specified, will be created to contain all the commands to execute each resource. This file can be modified and executed to build the proteome without the need for startup and configuration files. It is useful for debugging.

build_script_only

A 'YES' or 'NO' to indicate if only the master script should be created without running the pipeline.

log_file

A directory path and a name for the log file. A log file containing program execution details (including errors if they occur) is created.

email_url

E-mail address. An e-mail will be sent either to indicate that the proteome was built successfully OR failed. A log file is attached to the e-mail.

```
[Main]
work_dir="$HOME/vacceed"
species_dir="toxoplasma"
master_script="master_script"
build_script_only="NO"
log_file="$work_dir/$species_dir_logfile.txt"
email_url=Fred.Bloggs@student.uts.edu.au
```

Figure 10: Main – extract from a species configuration file

The keys under the Variables header (see Figure 11) are essentially used to save on typing and limit the number of changes required to the configuration file. The user can add any number of variables.

```
[Variables]
proteome_fasta="test.fasta"
prot_id_prefix="sp"
proteome_dir="$work_dir/$species_dir/proteome"
common_dir="$work_dir/$species_dir/pipeline/common_programs"
evidence_dir="$work_dir/$species_dir/pipeline/evidence/output"
resource_dir="[Resources.name]" # do not change
```

Figure 11: Variables – extract from a species configuration file

Description of keys under Variables header:

proteome_fasta = filename containing protein sequences from the target pathogen

prot_id_prefix = characters that precede the protein ID in the FASTA file specified with proteome_fasta key. For example, 'sp' for >sp|QQAAA| or 'tr' for >tr|QQBBB|. Note that the various descriptions in a FASTA definition are separated by the character '|'. Do not enter this character here.

proteome_dir = Path to directory containing the file assign by the key 'proteome_fasta'

common_dir = Path to directory containing programs that are common to more than one resource

evidence_dir = Path to directory that will contain the evidence files generated by the prediction resources.

resource_dir = value for this key must not change. This is a special case in which $resource_dir is replaced by the relevant resource name e.g. wolf.

Each resource has a possible four sections defined by 4 headers – [<resourceName >], [<resourceName >_files], [<resourceName >_programs], and [<resourceName >_arguments]. The resourceName should be consistent with the name of the resource used under the [Resources] header. Figure 12 represents a typical configuration for a resource. The directory for each resource contains an identical structure of two directories, which by default are called output and scripts.

```
[resourceName]
prog_dir="$work_dir/$species_dir/pipeline/$resource_dir"
script_dir="$work_dir/$species_dir/pipeline/$resource_dir/scripts"
out_dir="$work_dir/$species_dir/pipeline/$resource_dir/output"

[resourceName_files]
train_file="possibe_train_file"

[resourceName _programs]
1="resource_script"

[resourceName _arguments]
1="$proteome_fasta $proteome_dir $script_dir $out_dir $common_dir $evidence_dir $prog_dir"
```

Figure 12: Resource Name – extract from a species configuration file

Description of keys under resourceName:

prog_dir

The path to the directory that contains the Linux script for the resource. Typically the resource directory

script_dir

The directory name that will contain Linux scripts to execute the various commands of the resource. A separate script is created for each split group of proteins e.g. script1, script2 where each script processes a subset of the total number of proteins. These scripts are run either in parallel or consecutively (see section on **Running scripts in parallel**). The scripts can be run independently and are useful for debugging.

out_dir

The directory name that will contain all output files generated from the resource (See section on **Output Files**)

The list of keys under the header resourceName_files is used to specify variables for filenames. These variables are used as arguments to the resource script. It is highly recommended that careful attention is made to checking filenames because they may be species-specific.

The programs to run for each resource are numerically listed under the header resourceName_programs. Only one Linux script is listed for each resource in the distribution

version of *Vacceed*. This script typically contains all commands required to run other scripts and/or programs associated with the resource (see **Resource Scripts**). There is no limited to the number of programs that can be listed under this header. Each program is executed in numerical order commencing from key ‘1’.

Each program listed under resourceName_programs requires a corresponding list of arguments under the header resourceName_arguments. For example, the arguments following key ‘1’ are associated with the program following key ‘1’ (e.g. resource_script). It is critical the argument variables are retained in the correct order.

Resource Scripts

In the distribution version of *Vacceed*, each resource has one main Linux script (typically named after the resource) that in many instances creates subordinate scripts for processing a subset of the total number of proteins (see **Running scripts in parallel**). These scripts are saved in the scripts directory (see Figure 8). Each script (e.g. script1) contains all the required commands to execute the resource and to extract relevant evidence for a particular protein characteristic. There is a set hierarchal structure for the execution of all *Vacceed* scripts e.g. Startup → master_script → resource_script → subordinate script. Any script can be run independently, which is ideal for debugging. Each main resource script is constructed from a generic format. Figure 13a and continued on 13b shows an example of this format. There are seven main sections in the script:

Get command-line arguments – the arguments are passed by startup via the master_script. The resource arguments to pass are read from the appropriate species configuration file (see section **Specifies configuration file**). The arguments constitute the variables (e.g. denoted by the prefix ‘$’) used throughout the rest of the script.

Hard coding – local variables are recommended to be added here. bg_mode and split_by are required to implement parallel processing of scripts (see section **Running scripts in parallel**).

Split the total number of proteins into subsets – this section divides the protein sequences into temporary files saved in the output directory for the purpose of processing the files in parallel. The number of proteins in each temporary file is determined by the total number of proteins to be processed divided by the number assigned to the ‘split_by’ variable. For example, if the total number of proteins is 5000 and split_by = 6, then five temporary files

containing 833 protein sequences and one file containing 835 are created. The number of temporary files is assigned to the variable ‘no_of_files’, which in effect is the same as ‘split_by’. The default value for ‘split_by’ is the number of CPUs.

#Main loop for writing scripts – the idea behind this section is create a subordinate script that encapsulates all the commands required to process and manipulate data for a given subset of proteins. The general pattern for each step or command to be performed is to add one line as a description of the step, and another line with the actual command. Each command line should also include ‘|| error_exit’. A generic function called error_exit is executed in the event of an error raised by the command. All errors are written to the log file. A generic script called ‘error_script’ is used write the error_exit function.

#Run the scripts – a generic script called ‘run_scripts’ is used to execute each script.

# Tidy up – this section merges the output from the individual scripts and deletes all other temporary files.

# Extract Evidence – where possible a generic Perl script called ‘get_evidence.pl’ is used to extract from the merged resource output the required evidence. By default the extracted evidence is saved in the evidence_dir directory (see **Adding a new resource**).

All generic scripts are located in common_programs directory (see **Directory structure following *Vacceed* installation**).

```
#!/bin/sh
#Resource used: Example
resource=example
short_name=ex

#Get command-line arguments
proteome_fasta=$1
prog_dir=$2
script_dir=$3
out_dir=$4
common_dir=$5
evidence_dir=$6

#Hard coding
bg_mode=1 # parallel processing: 1 = ON, 0 = OFF
split_by=`nproc` #default split value is number of CPUs
ex_arg1="example_1"

#Split the total number of proteins into subsets
echo "Splitting FASTA file for " $resource >> $LOG_FILE
perl $common_dir/split_fasta.pl $proteome_dir/$proteome_fasta $split_by $out_dir
no_of_files=$?
if [ $no_of_files == 0 ]; then
        exit 1
fi
#Main loop for writing scripts
for file_no in $(seq 1 $no_of_files)
do
        #Write the error function to script
        $common_dir/error_script "$file_no" "$script_dir"

        #Program section
        echo "echo Running file $file_no for $resource >> $LOG_FILE" >> $script_dir/script$file_no

        echo "script_step=\"executing program_x for $file_no\"" >> $script_dir/script$file_no
        echo "program_x $ex_arg1 >$out_dir/$short_name$file_no \
         2>> $LOG_FILE || error_exit" >> $script_dir/script$file_no
done
```

Figure 13a: Example Resource Script

```
#Run scripts
$common_dir/run_scripts "$resource" "$no_of_files" "$script_dir" "$bg_mode" || exit 1

#Tidy up
echo "Tidying up for " $resource >> $LOG_FILE
cat $out_dir/${short_name}* > $out_dir/out_${short_name} || exit 1
rm $out_dir/${short_name}* || exit 1
rm $out_dir/*.fasta || exit 1

#Extract Evidence
#hard coding for extract output format
input_file=$out_dir/out_${short_name}
split_char='\s+'
id_info='1,2,\|'
evd_headers=$short_name"_score,"$short_name"_annotation"

echo "Executing get_evidence.pl" >> $LOG_FILE
perl $prog_dir/get_evidence.pl $input_file $evidence_dir $short_name $split_char $id_info $evd_headers\
2>> $LOG_FILE || exit 1
```

Figure 13b: Example Resource Script

Output Files

The two most important output files are vaccine_candidates and vaccine_candidates.fasta, which are saved in the **proteome** directory (see **Directory structure following *Vacceed* installation**).

vaccine_candidates contains an ordered list in a table format of each known and predicted protein in the proteome of the target pathogen (see example extract below). The order is determine by a final score based on the average of the machine learning (ML) scores.

```
#ID,nn,knn,ada,nb,rf,svm,existence_score,average_ML_score
S8F9N7,1.000,1.000,1.000,1.000,1.000,1.000,0.500,1.000
S8GL31,1.000,1.000,1.000,1.000,1.000,1.000,0.400,1.000
chrla_new10,1.000,1.000,1.000,1.000,1.000,1.000,0.010,1.000
S8GKZ9,0.400,0.667,0.000,1.000,0.025,0.108,0.370,0.367
S8GL08,0.450,0.667,0.000,0.999,0.007,0.070,0.140,0.366
S8FF43,0.000,0.000,0.000,0.000,0.000,0.007,0.700,0.001
```

Where nn = Neural network, knn = *k*-nearest neighbour classifier, ada = Adaptive boosting, nb = Naive Bayes classifier, rf = Random forest, and svm = Support vector machines.

There is one protein per row. Each column represents an average probability score between 0 and 1. This score represents the likelihood or confidence level that the 'YES' for vaccine classification is correct. The R functions for adaptive boosting, random forest, SVM, and naive Bayes classifier support class-probabilities i.e. an estimated probability for each protein belonging to 'YES' and 'NO' classes. The output from the R functions for *k*-nearest neighbour classifier and neural network is only a binary 'YES' or 'NO'. The score from these latter algorithms used in vaccine_candidates is therefore an average frequency for YES vaccine candidacy. The average score for all machine learning (ML) scores is reported in the last column and this value determines the order that the proteins appear in the list. There is also column that indicates a probability score that the protein is 'real'. This probability score is extracted from proteome_info.txt (see **PART A – Output Files**).

vaccine_candidates.fasta contains the protein sequences of the predicted vaccine candidates in a FASTA format for only proteins that have an average ML score and existence score from vaccine_candidates greater than user-defined threshold values. The threshold scores are assigned to the ml_threshold and existence_threshold keys under EVIDENCE resource in the species configuration file (default value = 0.75).

It is recommended that both 'vaccine_candidates' and 'vaccine_candidates.fasta' be examined after startup has finished and no errors were detected. If these files are empty or have missing or unexpected data, the next recommended step is to review the files in the output directory of the evidence resource. This latter directory should contain an output file from each resource. If a particular resource output file is missing or not what you expected, then probing the files in the output directory of the resource in question may provide some clues to the source of the problem. Each resource can potentially generate many output files. The output filenames, a description of their contents, and the program that generates them is listed in the following table. In general, the files represent an audit trail for the various steps performed by each resource. For the most part, you do not normally need to be concerned with these output files unless there is a requirement to scrutinise the quality of each step.

Program Name	Output file name	Description of contents
**Resource = VALIDATE**		
check_prot_seq.pl	validate_<sequence name>	Sequences that contain invalid amino acid characters i.e. a character other than [ARNDBCEQZGHILKMFPSTWYV]
**Resource = WOLF**		
runWolfPsortSummary	out_wolf	Subcellular predictions
get_evidence_for_wolf.pl	wolf_evd	Extract of the 'extr' and 'plas' localisation scores (file found in **evidence/output** directory
**Resource = SIGNALP**		
signalp	out_signalp	Secretory signal peptide predictions
get_evidence.pl	signalp_evd	Extract of the 'D' column (file found in **evidence/output** directory
**Resource = TARGETP**		
targetp	out_targetp	Secretory signal peptide predictions
get_evidence.pl	targetp_evd	Extract of the 'SP' column (file found in **evidence/output** directory
**Resource = TMHMM**		
tmhmm	out_tmhmm	Transmembrane helices predictions
get_evidence.pl	tmhmm_evd	Extract of 'ExpAA, First60, and PredHel' values (file found in **evidence/output** directory
**Resource = PHOBIUS**		
phobius.pl	out_phobius	Transmembrane helices and secretory signal peptide predictions
get_evidence.pl	phobius_evd	Extract of 'TM and SP' columns (file found in **evidence/output** directory
**Resource = MHCI**		
compute_peptides (invokes predict_binding.py)	out_mhci	Peptide-MHC I binding predictions
extract_mhci_output.pl	extract_stats.txt	Summary of high-affinity peptides per protein
	allele_stats.txt	Frequency of MHC I alleles used
	peptide_stats.txt	Frequency of peptide sequences
	id_allele_stats.txt	Frequency of alleles per protein
	id_peptide_stats.txt	Frequency of peptide sequences per protein

	id_allele_peptide_stats.txt	Frequency of allele and peptide sequences per protein
	allele_peptide_stats.txt	Frequency of allele and peptide sequences
extract_stats.pl	summary_of_extract.txt	Global summary of the file 'extract_stats.txt'
mhci_ml.pl	mhci_ml.txt	Peptide-mhc binding scores for each MHC I allele per protein in a format required for machine learning (ml) algorithms.
get_predictors.pl	ml_predictors.R	MHC I alleles that determine the predictors for machine learning (ml) algorithms (written in R syntax).
mhci_wrapper.R	predictions.txt	Probability values from multiple runs of randomForest (a machine learning algorithm) executed by Rscript. The value is an indicator of the potential of a protein to be a vaccine candidate.
extract_predictions.pl	mhci_evd	Average vaccine candidacy probability values extracted from predictions.txt (file found in **evidence/output** directory
**Resource = MHCII**		
compute_peptides (invokes mhc_II_binding.py)	out_mhcii	Peptide-MHC II binding predictions
extract_mhcii_output.pl	extract_stats.txt	Summary of high-affinity peptides per protein
	allele_stats.txt	Frequency of MHC II alleles used
	peptide_stats.txt	Frequency of peptide sequences
	id_allele_stats.txt	Frequency of alleles per protein
	id_peptide_stats.txt	Frequency of peptide sequences per protein
	id_allele_peptide_stats.txt	Frequency of allele and peptide sequences per protein
	allele_peptide_stats.txt	Frequency of allele and peptide sequences
extract_stats.pl	summary_of_extract.txt	Global summary of the file 'extract_stats.txt'
mhcii_ml.pl	mhcii_ml.txt	Peptide-mhc binding scores for each MHC II allele per protein in a format

		required for machine learning (ml) algorithms.
get_predictors.pl	ml_predictors.R	MHC II alleles that determine the predictors for machine learning (ml) algorithms (written in R syntax).
mhcii_wrapper.R	predictions.txt	Probability values from multiple runs of randomForest (a machine learning algorithm) executed by Rscript. The value is an indicator of the potential of a protein to be a vaccine candidate.
extract_predictions.pl	mhcii_evd	Average vaccine candidacy probability values extracted from predictions.txt (file found in **evidence/output** directory)
**Resource = EVIDENCE**		
combine_evidence.pl	evidence_profiles	Merged content of all files with the suffix '_evd'. This file constitutes the input file for machine learning algorithms.
get_predictors.pl	ml_predictors.R	Evidence as defined by the column headers in 'evidence_profiles' that determine the predictors for machine learning (ml) algorithms (written in R syntax). Note: EVIDENCE key called ignore_predictors in species configuration file is used to list the headers to ignore as predictors.
<al>_wrapper.R<br><al>_runPred.R<br><al>_makePred.R (can change algorithm parameters in this file)	<al>_predictions.txt	Probability values from multiple runs of a machine learning algorithm executed by Rscript. The value is an indicator of the potential of a protein to be a vaccine candidate. <al> represents a character prefix to denote the algorithm used: ada = Adaptive boosting, rf = Random forest, knn = *k*-nearest neighbour classifier, nb = Naive Bayes classifier, nn = Neural network, svm = Support vector machines
extract_prob_predictions.pl	<al>_ml	Average probability per protein for YES vaccine candidacy as extracted from

		<al>_predictions.txt. Only applicable to the algorithms that output class probabilities e.g. ada, rf, svm, and nb
extract_class_predictions.pl	<al>_ml	Average frequency per protein for YES vaccine candidacy as extracted from <al>_predictions.txt. Only applicable to the algorithms that output a binary YES or NO e.g. nn
extract_knn_predictions.pl	<al>_ml	Probability per protein for YES vaccine candidacy as extracted from knn_predictions.txt. Only applicable to the algorithm knn
combine_scores.pl	vaccine_candidates	Includes for each protein: score from each machine learning (ml) algorithm; score for protein existence; average ml score (1= maximum and 0= minimum confidence in prediction) (file found in **proteome** directory)
get_candidate_fasta.pl	vaccine_candidates.fasta	Contains the protein sequences in a FASTA format for only proteins that have an average ML score and existence score from vaccine_candidates greater than user-defined threshold values. Threshold scores are assigned to the ml_threshold and existence_threshold keys under EVIDENCE resource in the species configuration file (default value = 0.75) (file found in **proteome** directory)

Running scripts in parallel

The resources encapsulate, for the most part, a large number of independent computation-intensive tasks. *Vacceed* takes advantage of multi-core processors. The default when running the pipeline is to divide protein sequences into a number of temporary files for the purpose of processing the files in parallel. The number of proteins in each temporary file is determined by the total number of proteins to be processed divided by the number assigned to the 'split_by' variable in the resource script (see **Resource Scripts**). For example, if the total number of proteins is 5000 and the split_by = 6, then five temporary files containing 833

protein sequences and one file containing 835 are created. The default value for ‘split_by’ is the number of CPUs but you can override this by assigning the desired split number to ‘split_by’. The temporary files can be run in parallel or consecutively depending on the setting for bg_mode (default is bg_mode=1 for parallel processing – see **Resource Scripts**). If there are more temporary files than CPUs, then the surplus files are queued i.e. when a file has finished processing a new one will commence.

Adding a new resource

This section describes the steps required to add a new resource. It is assumed here that the resource is to contain a fictitious program called program_z that predicts a particular protein characteristic. The primary goal is to extract relevant evidence from the output of program_z to add to overall evidence profile for the purpose of vaccine candidacy decision making using machine learning algorithms.

1. Install program_z and append program location to the PATH variable so that the program will run from any directory. The best place to add the location is to modify the user's .bash_profile file.
   E.g. *PATH=$PATH:$HOME/Pipeline_Programs/program_z/bin*

2. Test that the program will run with sample data and ensure it can be invoked from any directory. Furthermore, determine the input and output requirements.

3. Add a new resource name in the appropriate configuration file for running the pipeline.

   [Resources]
   name=VALIDATE,WOLF,TMHMM,TARGETP,PHOBIUS,**NEW_RESOURCE**,EVIDENCE

4. Add a new section to the same configuration file. The easiest way to do this is to copy an existing resource and amend accordingly. The texts highlighted in red are the only parts expected to be changed (see **Species configuration file** for more details).

   [NEW_RESOURCE] ← This must be the same name as that used in step 3.
   prog_dir="$work_dir/$species_dir/pipeline/$resource_dir"
   script_dir="$work_dir/$species_dir/pipeline/$resource_dir/scripts"
   out_dir="$work_dir/$species_dir/pipeline/$resource_dir/output"

   [NEW_RESOURCE_files]

```
train_file="possibe_train_file" ← A training file may not be required for some programs
additional_file="file.txt" ← Only if required

[NEW_RESOURCE_programs]
1="new_resource_script"

[NEW_RESOURCE_arguments]
1="$proteome_fasta $proteome_dir $script_dir $out_dir $evidence_dir $prog_dir"
```

5. Create a new directory in the ‘pipeline’ (see **Directory structure following *Vacceed* installation)** using the same name (but in lowercase) as the new resource; then create two directories called ‘output’, and ‘scripts’ in this newly created directory.

6. Copy the ‘template_resource_script’ from the common_programs directory into the new resource directory and rename it to the same name as that specified for the program under [NEW_RESORCES_programs] header.

7. Amend the new_resource_script from step 6 (see **Resource Scripts** for example script). Add the new program or programs within the ‘Main loop for writing scripts’ where is states “<< Add new programs here >>”. You need to ensure that the required arguments (i.e. the ‘inputs’ as determined in step 2) are passed to program_z and the output is to $out_dir

```
#Step description
echo "script_step=\">> executing program_z\"" >> $script_dir/script$chr_no
echo "program_z $required_input $out_dir" >> $script_dir/script$chr_no \
|| error_exit
```

8. The next step is totally dependent on the output generated by program_z. Here, we need to extract the relevant evidence from the output file. There is a generic Perl script in the common_programs directory called ‘get_evidence.pl’ that potentially can be used. This script can be used in the following scenarios.

i) When the output is presented in columns delimited by a fixed character.

E.g.

```
ID              col2    col3    col4    col5
sp|rr|045|A4    0.5     2.4     6.2     8.9
This is a line that should be ignored
sp|rr|099|A4    0.8     3.9     7.2     10.2
Another line to ignore
```

Let us say for example that you need to extract the data from column 2 and column 5. Amend new_resource_script as follows:

```
Short_name='new_res'
input_file=$out_dir/out_${short_name} <- path and name of output file
split_char='\s+' <- A fixed character that separates the columns e.g. ',' or ':' or '|'. In this case a Perl regular expression is used for one of more white spaces between columns
cols_required='2,5'  <- List of column numbers to extract
id_info='1,3,\|' <- Comma delimited information for extracting ID e.g. the ID '045' from sp|045|A4  where 1 = the column in the input line containing the identifier (e.g. 1), 2 = the position of the ID in the identifier (e.g. 3), 3 = the separating character between parts of the identifier (e.g. |)  (Note that a '\' is used to escape the character.
evd_headers=$short_name"_score,"$short_name"_validate" <- name of headers to use in evidence profile
start_prefix='sp' <- Determines which lines to extract the data columns from. In this case all line starting with 'sp'
ignore_lines='This is a, Another' <- a comma delimited list of text. Any lines that commence with the same text will be ignored

echo "Executing get_evidence.pl" >> $LOG_FILE
perl $prog_dir/get_evidence.pl $input_file $evidence_dir $short_name $split_char \
$cols_required $id_info  $evd_headers $start_prefix $ignore_lines 2>> $LOG_FILE || exit 1
```

ii) When the output is presented in columns delimited by a fixed character AND the column header prefixes each value

E.g.

```
ID=sp|rr|045|A4,score=0.5,prob=2.4,percent=6.2,validate=8.9
ID=sp|rr|099|A4,score=0.8,prob=3.9,percent=7.2,validate=10.2
```

Let us say for example that you need to extract the data from column 2 and column 5. Amend new_resource_script as follows:

```
Short<name='new_res'
input_file=$out_dir/out_${short_name}
split_char=',' <- In this case a comma
cols_required='2,5'
id_info='1,3,\|'
evd_headers=$short_name"_score,"$short_name"_validate"
extract_value='=' <- a fixed character that separates the column header from the value. In this case an '='
```

```
echo "Executing get_evidence.pl" >> $LOG_FILE
perl $prog_dir/get_evidence.pl $input_file $evidence_dir $short_name $split_char $id_info \
$cols_required $evd_headers  $extract_value 2>> $LOG_FILE || exit 1
```

9. Provided you have added a program that extracts the relevant output and saves it in a file with the suffix '_evd' in the evidence/output directory, no further amendments or steps are required.

Appendix A

Introduction

The current trend in vaccine development is epitope-based due to its potential to be more specific, safer, and easier to produce than traditional vaccines [1]. The key to subunit vaccine development is the successful identification of proteins of a pathogen, as opposed to using the entire entity, which evoke a protective, safe immune response. Proteins that are present on the surface of the pathogen or are secreted from the pathogen are the most likely candidates to induce an immune response and are consequently the target for this study. Five programs (WoLF PSORT [2], SignalP [3], TargetP [4], TMHMM [5], and Phobius [6]) were used to predict protein characteristics relevant to sub-cellular location given amino acid sequences as input.

It is the recognition of epitopes on pathogens by T- and B-cells (and soluble antibodies) that activates the cellular and humoral immune response [7]. The premise here is that if a high affinity epitope can be associated with a protein then this provides further evidence for the protein's vaccine candidacy. Two programs (MHC I Binding Predictor and MHC II Binding Predictor [8, 9]) were used to predict peptide binding to MHC class I and class II molecules.

All seven programs were essentially chosen because they were applicable to eukaryotes, could be freely downloaded, run in a standalone mode, allow high throughput processing, and execute in a Linux environment. All programs can be executed via web interfaces. However, processing enormous amounts of input is currently unproductive through web interfaces and in particular the web versions of WoLF PSORT, SignalP, and TargetP restrict the number of input sequences; hence the reason why equivalent standalone versions of the programs were employed here in a Linux environment.

WoLF PSORT

WoLF PSORT computationally predicts a protein's localization and in effect mimics the biological mechanism of protein sorting [10] by which a protein, after its encoding and synthesis, is transported to the appropriate position in or outside the pathogen. The main determinant of a protein's localization is the protein amino acid sequence [2]. In effect the sequence contains a delivery address. Many programs have been developed to predict

subcellular locations of proteins [4, 11][1]. Most programs are web based. The prediction methods can be broadly grouped into two classifications: rules/knowledge based and machine learning. The rules based method exploits static knowledge of what determines subcellular location, whereas the machine learning method dynamically utilises training data to identify subcellular locations by focusing on the differences between proteins from different known locations. PSORT [12] is a well-known, well-used example; PSORT II [12] is both rules and machine learning based.

WoLF PSORT is an extension of the PSORT II program and can be used for the prediction of protein localisation sites in eukaryotic pathogens [2]. It requires as input, full-length amino acid sequences of a protein in a FASTA format. The program detects sorting signals within the sequence from which it then computationally predicts the protein's subcellular localisation. Signal detection is achieved by applying stored rules for various sequence features with specific criteria of known sorting signal motifs e.g. the feature is a GPI-anchor and the criteria for the feature is 'type-1a membrane protein with a very short tail'. One of the extensions to PSORT II implemented by WoLF PSORT is the identification of localisation features using amino acid composition and functional motifs such as DNA-binding motifs. A weighted *k*-nearest-neighbour classifier estimates the likelihood of localization features being sorted to each candidate site (referred in the program as a localisation class) and outputs the most probable sites with a score. A training dataset is required comprising protein sequences with a known localisation label. The training set supplied with the program is stated to be applicable to animals and contains 12,000 UniProt sequences [2].

Figure A1 shows a typical output from WoLF PSORT. Information about each protein sequence is displayed on separate lines (only three sequences are shown in Figure A1). Each field along the line contains a localization class (based on Uniprot "Subcellular Localization" field keywords) and a score separated by a comma. There are 12 localisation classes that also map to Gene Ontology (GO)[2].

```
seq1 extr_plas: 11.5, plas: 11, extr: 10, E.R.: 4, lyso: 4, pero: 1.5
seq2 extr: 25, lyso: 3, plas: 2, nucl: 1, E.R.: 1
seq3 extr: 31, lyso: 1
```

Figure A1. Typical output from WoLF PSORT

[1] Subcellular location prediction programs at http://www.ncbi.nlm.nih.gov/CBBresearch/Lu/subcellular/

[2] Gene Ontology (GO) website at: http://www.geneontology.org/

As an example of how to interpret the output in Figure A1 protein ‘seq1’ has six candidate sites listed in descending order of likelihood based on a score. The most likely site is extracellular (extr) and plasma membrane (plas) i.e. there is dual localisation with a score of 11.5. The plasma membrane (on its own) is the next most likely site, followed by extracellular, endoplasmic reticulum (E.R.), lysozyme (lyso) and finally peroxisome (pero). The accuracy of WoLF PSORT is influenced by the number of each type of localisation site in the training data .e.g. sites with few examples in the training dataset are seldom correctly predicted.

SignalP

One of the most well-known protein sorting signals is the secretory signal peptide, which targets its passenger protein to the secretory pathway via the endoplasmic reticulum. The secretory pathway is a series of steps that ends with the secretion of a protein through the cell plasma membrane to the outside of the pathogen. It is important to know that not all secretory proteins have signal peptides, or are necessarily secreted to the outside of the pathogen [4]. Some proteins have specific retention signals that hold them back in the ER or the Golgi or divert them to the lysosomes [4]. There are many different types of secretory signal peptides but the most common type is the signal peptide cleaved by signal peptidase. Although there are no simple consensus sequences, three distinct compositional regions on the peptide’s amino acid sequence help define this type of signal peptide: N-terminal, central hydrophobic, and C-terminal regions. Specific motifs that target the protein are within the N-terminal, and the signal peptidase cleavage site that precedes the mature protein is within the C-terminal. Signal peptides are cleaved off while the protein is translocated through the cell membrane [13]. There are also signal peptides that are not cleaved called signal anchors i.e. a transmembrane protein with one transmembrane segment near the N-terminal of the protein [14, 15].

Secretory signal peptides can be computationally predicted using machine learning techniques, such as neural networks and hidden Markov models (HMMs) [16]. The program SignalP (version 4.0) predicts the presence and location of the signal peptidase I cleavage site at the C-terminal end of the presequence; and classifies each residue in the presequence as either belonging or not belonging to a signal peptide using two neural networks. Two different types of negative data were used for training the neural network models: sequences (mostly derived from UniProt) with transmembrane regions located within the first 70 residues from the N-terminal were used to train the SignalP-TM network; and sequences from non-secretory proteins were used to train the SignalP-noTM network. If the network SignalP-

TM predicts four or more positions as transmembrane positions, SignalP-TM is used for the final prediction, otherwise SignalPnoTM is used. Training data for eukaryotes is supplied with SignalP.

The input format required is multi-FASTA. It is recommended in the SignalP user manual that only the first 50 to 70 amino acids[3] of each sequence should be used in the prediction as longer sequences increase the risk of false positives. To restrict the length of the input sequence a command-line parameter is used (e.g. –trunc 70). An example of the summary output from SignalP is shown in Figure A2.

SignalP comprises five different scores between 0 and 1: 1) *Cmax* is the maximum "cleavage site"' score (a C-score is calculated for each position in the submitted sequence and a significant high score indicates a cleavage site); 2) *Ymax* is a derivative of the *C-score* combined with the S-score resulting in a better cleavage site prediction than the raw *C-score* alone. 3) S-max is the "maximum signal peptide" prediction score (the *S-score* for the signal peptide prediction is calculated for every single amino acid position in the submitted sequence and a high score indicates that the corresponding amino acid is part of a signal peptide, and a low score indicates that the amino acid is part of a mature protein); 4) Smean is the "average of the S-score", and 5) D is an average of the "Smean and Ymax" score. Position (pos) is the location in the amino acid sequence where Cmax (.i.e. cleavage site position), Ymax (i.e. length of signal peptide), and Smax occur. The "Y" or "N" is a yes or no indication that the sequence has a cleavage site and a signal peptide, when D is above or below the Dmaxcut. High scores also indicate that the sequence is a secretory protein. According to the authors of SignalP, a high D-score is the best indicator of secretory proteins [14].

```
# SignalP-4.0 euk predictions
# name    Cmax  pos  Ymax  pos  Smax  pos  Smean   D     ?  Dmaxcut    Networks-used
Q9UB12  0.174  31  0.302  22  0.794  14  0.660   0.495 Y  0.450      SignalP-noTM
Q58L79  0.229  49  0.234  49  0.416  48  0.193   0.218 N  0.500      SignalP-TM
Q9GU48  0.775  24  0.817  24  0.945  13  0.862   0.841 Y  0.450      SignalP-noTM
```

Figure A2. Typical summary output format from SignalP

TargetP

TargetP is similar to SignalP. Neural networks are also implemented to predict subcellular locations of eukaryotic protein sequences. More specifically, TargetP predicts the presence and length of secretory pathway signal peptides (SP) and mitochondrial targeting peptides (mTP) in the N-terminal presequences [17]. As with SignalP the input is a protein sequence in a FASTA format. An example of TargetP output is shown in Figure A3. Len is the sequence

[3] N-terminal peptides typically comprise 15-30 amino acids

length, followed by neural network scores for mitochondrial targeting peptide (mTP), secretory signal peptide (SP), and "other" localizations. The predicted localisation (loc) based on the scores is either mitochondrion (M) or secretory pathway (S) or any other location (-). The reliability class (RC) is from 1 (most reliable) to 5 (least reliable) and is a measure of prediction certainty. The truncated peptide length (TPlen) indicates the predicted presequence length to the cleavage site.

```
### targetp v1.1 prediction results ##########################
Number of query sequences:  3
Cleavage site predictions included.
Using OTHER networks.

Name                  Len     mTP      SP  other  Loc  RC  TPlen
----------------------------------------------------------------
Seq_1                  97   0.555  0.014  0.150   M     5   40
Seq_2                1088   0.070  0.067  0.822   _     2    -
Seq_3                 117   0.095  0.967  0.006   S     1   26
```

Figure A3. Typical output format from TargetP v1.1

TMHMM

Being exposed to the outside environment, surface membranes of pathogens are in full view of a host's immune system surveillance. Consequently membrane molecules, including proteins spanning or anchored to the membrane, are likely to be antigenic. A transmembrane protein that spans an entire membrane has predominantly a hydrophobic domain consisting of one or multiple α-helices motifs [5]. Numerous programs to predict transmembrane helices have been developed over the last 30 years — programs such as DAS, SOSUI, SPLIT, TMAP, TMpred, TopPred 2, MEMSAT, HMMTOP, ALOM 2 and Tmpro. Moller and colleagues evaluated methods for the prediction of membrane spanning regions [18]. Most prediction methods are based on hydrophobicity of amino acid residues and/or the abundance of positively charged residues on the cytoplasmic side of the membrane and/or the protein's topology patterns of cytoplasmic and non-cytoplasmic loops. These methods are applicable for almost all organisms [5]. The majority of programs are web servers and therefore are unsuitable for high-throughput processing.

The program TMHMM based on a hidden Markov model approach [5] predicts transmembrane helices in given protein sequences in a FASTA format. Figure A4 shows one line of a typical output from TMHMM in a summary format. Each output line shows the length (len) of the protein sequence followed by the expected number of amino acid residues in transmembrane helices (ExpAA). If the ExpAA number is larger than 18 (a value proposed

by the TMHMM creators) it is very likely to be a transmembrane protein (or have a signal peptide). The output line also shows the expected number of residues in the transmembrane helices in the first 60 amino acids of the protein (First60), the number of predicted transmembrane helices (PredHel), and the predicted protein topology i.e. the in/out orientation of the protein relative to the membrane. TMHMM occasionally incorrectly predicts a transmembrane helix in the N-terminal region when it is a signal peptide. This prediction error is mainly because signal peptides also contain a hydrophobic region. The larger the First60 number the more likely the predicted transmembrane helix in the N-terminal is a signal peptide. The creators of THHMM propose that a First60 value greater than 10 indicates a possible N-terminal signal sequence.

```
Seq_1 len=278 ExpAA=68.69 First60=39.89 PredHel=3 Topology=i7-29o44-66i87-109o
```

Figure A4. Typical summary output format from TMHMM v2.0

Phobius

An evaluation of signal sequence prediction methods conducted by Menne and colleagues indicated that SignalP was more sensitive than other methods but included many false positive predictions [19]. An inherent problem in signal peptide prediction is the high similarity between the hydrophobic regions of a transmembrane (TM) helix and that of a signal peptide (SP) can result in a TM helix falsely classified as a SP, or conversely a SP falsely classified as a TM helix [15]. Phobius is a combined transmembrane domain and signal peptide predictor that can help discriminate between TM helices and SPs and also add endorsement to TMHMM predictions. The different sequence regions of a signal peptide and a transmembrane protein are modelled in Phobius with hidden Markov models. Figure A5 shows the output from Phobius in a short format. The output information for one protein sequence (SEQENCE) per line consists of the number of transmembrane (TM) helices, a “Y” or “N” indicator that the sequence has a signal peptide (SP), and a predicted topology (information for only one protein sequence is shown).

```
SEQENCE   TM SP PREDICTION
Seq_1      7 Y n4-19c24/25o219-238i250-269o281-302i322-342o372-391i422-439o451-476i
```

Figure A5. Typical short output format from Phobius

MHC Binding Predictors

One of the foremost resources for T-Cell MHC class I and II binding prediction tools is provided by the Immune Epitope Database Analysis Resource (IEDB). IEDB provides a download Linux package (for a 32 bit system) that contains a collection of peptide binding prediction tools for MHC class I and class II molecules. Included in the package are NetMHCpan and NetMHCIIpan, which are extended versions to NetMHC and netMHCII. The collection of tools is a mixture of Python scripts and Linux specific binary files. Python 2.5 or higher is therefore a prerequisite to run the tools. These tools take as input an amino acid sequence (or a set of sequences) and determine the ability of each subsequence to bind to a specific MHC molecule. For MHC class I the available prediction methods are: artificial neural network (ANN) [20], Average relative binding (ARB) [21], Stabilized matrix method (SMM) [22], SMM with a Peptide-MHC Binding Energy Covariance matrix (SMMPMBEC), Scoring Matrices derived from Combinatorial Peptide Libraries (Comblib_Sidney2008) [23], Consensus [24], and NetMHCpan [25]. A large scale evaluation of three MHC class I binding prediction methods (ANN, SMM, and ARB) was conducted in 2006 [26]. Each of the three methods predicts the quantitative affinity of a peptide for an MHC molecule. In the evaluation, the predicted affinities of all three methods were compared to a collection of experimentally measured peptide affinities to MHC class I molecules. Linear correlation coefficients were calculated between predicted and measured affinities on a logarithmic scale. The evaluation reported that ANN preformed the best in a statistically significant manner (with a correlation coefficient of 0.69), followed by SMM (0.62) and then ARB (0.55) [26].
The MHC II binding peptide predictions are more computationally challenging than for MHC class I and this seems to be reflected in the inferior prediction performance of class II algorithms in comparison to those in class I [21]. In the IEDB download package, the available prediction methods for MHC class II are: Consensus [27], Average relative binding (ARB) [21], combinatorial library (unpublished method), NN-align [28] (this method is the equivalent to netMHCII version 2.2), SMM-align [29] (equivalent to netMHCII version 1.1), Sturniolo [30] (a method also used in the program TEPITOPE [31]), and NetMHCIIpan [32]. Wang and colleagues assessed MHC class II peptide binding prediction methods in 2008 [27]. The IEDB curators rank Consensus as the best method, followed by NN-align, SMM-align, combinatorial library, Sturniolo, and ARB.

The performance of a prediction method is governed by the availability of MHC alleles. In other words, not all methods can currently make predictions for all MHC allele and peptide length combinations (e.g. there may be insufficient experimental data available to generate the combinations). The Consensus method is recommended by the creators because this

method consecutively uses several prediction methods. For example, for each MHC I allele and peptide length combination ANN method is tried first, SMM is tried next, and then comblib_Sidney2008, ARB, and finally NetMHCpan is tried if no previous method was available for the allele-length combination.

Prediction methods are encapsulated in two programs: predict_binding for MHC class I and mhc_II_binding for MHC class II. The method to use in the prediction is given as a command-line parameter. Figure A6 shows an example of the command line syntax. Only one MHC allele is analysed at a time. Therefore, the relevant program needs to be executed multiple times for each possible MHC allele-peptide length combination.

Figure A7 shows a typical output from the MHC class I predictor using a Consensus method (some columns have been deleted and the format adjusted to fit output on the page). Beginning at the start amino acid (numbered 1) of each sequence (denoted by #), a test subsequence of a specific peptide length (e.g. PepLengh = 9) is created (e.g. Sequence = MSMEGDRPS and is located from amino acids 1 to 9 on sequence input #1). The subsequence is scored (e.g. in units of IC_{50}nM) for binding affinity against the MHC allele e.g. HLA-A*02:05, using different prediction methods scores are calculated for each amino acid at each position in the subsequence, which are then added to yield the overall binding affinity.

```
## Command-line syntax For MHC I ##
predict_binding.py arb "HLA-A*02:01" 9 sequence.fasta
# arb = ARB prediction method
# "HLA-A*02:01" = MHC allele name
# 9 = peptide length
# sequence.fasta = name of file containing amino acid sequences in a FASTA format

## Command-line syntax For MHC II ##
mhc_II_binding.py consensus3 HLA-DRB1*03:01 sequence.fasta
# consensus = consensus prediction method
# HLA-DRB1*03:01 = MHC allele name
# sequence.fasta = name of file containing amino acid sequences in a FASTA format
```

Figure A6. Command-line syntax for executing IEDB MHC binding peptide predictor

In the example Figure 7A, method NetMHCpan was used because no previous method was available for the allele-length combination. However, the output could in theory contain scores from multiple methods if the method was available for the allele-length combination. The next test subsequence in Figure 7A is “SMEGDRPSG” from amino acids 2 to 10 on sequence input #1 and is scored against the same MHC allele, and so on. The affinity of the MHC allele and subsequence binding is greater the lower the IC_{50} value. The program

creators propose a rough guideline for interpretation: peptides with IC_{50} values <50 nM are considered high affinity, <500 nM intermediate affinity, and <5000 nM low affinity.

Allele	#	Start	End	PepLength	Sequence	Method	IC50(nM)
HLA-A*02:05	1	1	9	9	MSMEGDRPS	NetMHCpan	6829.04
HLA-A*02:05	1	2	10	9	SMEGDRPSG	NetMHCpan	26123.53
HLA-A*02:05	1	3	11	9	MEGDRPSGA	NetMHCpan	3.32

Figure A7. Typical output from IEDB MHC I peptide binding predictor

As per Figure 7A there are multiple peptide affinity scores for each protein. The purpose of using the IEDB epitope prediction was to gather further evidence of a protein's vaccine candidacy rather than to identify specific epitopes for vaccine development. Ideally, a single score was required to encapsulate the collective potential of the epitopes on a protein antigen. In *Vacceed*, random forest (a machine learning algorithm) is used to predict a single probability value that a protein had vaccine candidacy potential.

References

1. Zhao B, Sakharkar K, Lim C et al. MHC-Peptide binding prediction for epitope based vaccine design, International Journal of Integrative Biology 2007;1.
2. Horton P, Park K-J, Obayashi T et al. WoLF PSORT: protein localization predictor, Nucleic Acids Research 2007;35:W585-W587.
3. Petersen TN, Brunak S, von Heijne G et al. SignalP 4.0: discriminating signal peptides from transmembrane regions, Nature Methods 2011;8:785-786.
4. Emanuelsson O, Brunak S, von Heijne G et al. Locating proteins in the cell using TargetP, SignalP and related tools, Nat. Protocols 2007;2:953-971.
5. Krogh A, Larsson B, von Heijne G et al. Predicting transmembrane protein topology with a hidden markov model: application to complete genomes, Journal of Molecular Biology 2001;305:567-580.
6. Kall L, Krogh A, Sonnhammer ELL. A combined transmembrane topology and signal peptide prediction method, Journal of Molecular Biology 2004;338:1027-1036.
7. Flower DR, Macdonald IK, Ramakrishnan K et al. Computer aided selection of candidate vaccine antigens, Immunome Research 2010;6 Suppl 2:S1.
8. Kim Y, Ponomarenko J, Zhu Z et al. Immune epitope database analysis resource, Nucleic Acids Research 2012;40:W525-W530.
9. Kim Y, Sette A, Peters B. Applications for T-cell epitope queries and tools in the Immune Epitope Database and Analysis Resource, Journal of Immunological Methods 2011;374:62-69.
10. Richards JE, Hawley RS. The Central Dogma of Molecular Biology: How Cells Orchestrate the Use of Genetic Information. The Human Genome (Third Edition). San Diego: Academic Press, 2011, 83-113.
11. Emanuelsson O. Predicting protein subcellular localisation from amino acid sequence information, Briefings in Bioinformatics 2002;3:361-376.
12. Nakai K, Horton P. PSORT: a program for detecting sorting signals in proteins and predicting their subcellular localization, Trends in Biochemical Sciences 1999;24:34-35.

13. von Heijne G. Signal Peptides. eLS. John Wiley & Sons, Ltd, 2001.
14. Dyrløv Bendtsen J, Nielsen H, von Heijne G et al. Improved Prediction of Signal Peptides: SignalP 3.0, Journal of Molecular Biology 2004;340:783-795.
15. Nielsen H, Engelbrecht J, Brunak S et al. Identification of prokaryotic and eukaryotic signal peptides and prediction of their cleavage sites, Protein Engineering 1997;10:1-6.
16. Nielsen H, Brunak S, von Heijne G. Machine learning approaches for the prediction of signal peptides and other protein sorting signals, Protein Engineering 1999;12:3-9.
17. Emanuelsson O, Nielsen H, Brunak S et al. Predicting Subcellular Localization of Proteins Based on their N-terminal Amino Acid Sequence, Journal of Molecular Biology 2000;300:1005-1016.
18. Möller S, Croning MDR, Apweiler R. Evaluation of methods for the prediction of membrane spanning regions, Bioinformatics 2001;17:646-653.
19. Menne KML, Hermjakob H, Apweiler R. A comparison of signal sequence prediction methods using a test set of signal peptides, Bioinformatics 2000;16:741-742.
20. Nielsen M, Lundegaard C, Worning P et al. Reliable prediction of T-cell epitopes using neural networks with novel sequence representations, Protein Science 2003;12:1007-1017.
21. Bui H-H, Sidney J, Peters B et al. Automated generation and evaluation of specific MHC binding predictive tools: ARB matrix applications, Immunogenetics 2005;57:304-314.
22. Peters B, Sette A. Generating quantitative models describing the sequence specificity of biological processes with the stabilized matrix method, BMC Bioinformatics 2005;6:132.
23. Sidney J, Assarsson E, Moore C et al. Quantitative peptide binding motifs for 19 human and mouse MHC class I molecules derived using positional scanning combinatorial peptide libraries, Immunome Research 2008;4:2.
24. Moutaftsi M, Peters B, Pasquetto V et al. A consensus epitope prediction approach identifies the breadth of murine TCD8+-cell responses to vaccinia virus, Nature biotechnology 2006;24:817-819.
25. Hoof I, Peters B, Sidney J et al. NetMHCpan, a method for MHC class I binding prediction beyond humans, Immunogenetics 2009;61:1-13.
26. Peters B, Bui H-H, Frankild S et al. A community resource benchmarking predictions of peptide binding to MHC-I molecules, PLoS Computational Biology 2006;2:574-584.
27. Wang P, Sidney J, Dow C et al. A systematic assessment of MHC class II peptide binding predictions and evaluation of a consensus approach, PLoS Computational Biology 2008;4.
28. Nielsen M, Lund O. NN-align. An artificial neural network-based alignment algorithm for MHC class II peptide binding prediction, BMC Bioinformatics 2009;10.
29. Nielsen M, Lundegaard C, Lund O. Prediction of MHC class II binding affinity using SMM-align, a novel stabilization matrix alignment method, BMC Bioinformatics 2007;8:238.
30. Sturniolo T, Bono E, Ding JY et al. Generation of tissue-specific and promiscuous HLA ligand databases using DNA microarrays and virtual HLA class II matrices, Nature biotechnology 1999;17:555-561.
31. Bian HJ, Hammer J. Discovery of promiscuous HLA-II-restricted T cell epitopes with TEPITOPE, Methods 2004;34:468-475.
32. Nielsen M, Justesen S, Lund O et al. NetMHCIIpan-2.0 - Improved pan-specific HLA-DR predictions using a novel concurrent alignment and weight optimization training procedure, Immunome Research 2010;6:9.
